# Supplementary material for: Uptake of COVID-19 vaccines and associated factors among adults in Uganda: a cross-sectional survey
Source: BMJ Open. 2023 Mar 17;13(3):e067377. doi: 10.1136/bmjopen-2022-067377 (PMC10030279; doi:10.1136/bmjopen-2022-067377)
Supplement: Supplementary data [file bmjopen-2022-067377supp001.pdf]

Uptake of COVID-19 vaccines and associated factors among adults in Uganda: a cross-sectional survey

Question	Answer	
MAKERERE SCHOOL OF PUBLIC HEALTH AND MIT GOV/LAB MOBILE SURVEY QUESTIONNAIRE		
Phone number used for interview		
Did the call go through?	1	Yes
	0	No
	-97	DO NOT READ: Refused
Was this the respondent previously interviewed?	1	Yes
	0	No
	-97	DO NOT READ: Refused
[Insert Consent Text]		
Do you voluntarily agree to participate in this survey?	1	Yes
	0	No
	-97	DO NOT READ: Refused
Explain:		
A7. What is your name?		
A8. What is your age?		
A9. What region do you live in?	1	North
	2	East
	3	Central
	4	West
	-97	DO NOT READ: Refused
A10. What district do you live in?	[Insert filtered list of districts]	
A11. Do you live in an urban, rural, or semi-urban area?	1	Urban
	2	Rural
	3	Semi-urban
	-99	DO NOT READ: Don't know
	-97	DO NOT READ: Refused
A12. Gender of respondent	1	Female
	0	Male
Next, I am going to ask some questions on your awareness and intention to take the COVID-19 vaccine.		
E1. As an adult, have you received a vaccine other than the COVID-19 vaccines? (Hepatitis B, Yellow fever, tetanus)	1	Yes
	0	No
	-97	DO NOT READ: Refused
E2. Have you ever been asked to get a vaccine and declined?	1	Yes
	0	No
	-97	DO NOT READ: Refused

Question	Code	Answer
E3. What COVID-19 vaccines have you heard of?<br>*Do not read options aloud. Select all that apply.*	1	AstraZeneca
	2	Pfizer-BioNTEch
	3	Moderna
	4	Johnson and Johnson
	5	Sinopharm
	6	Sinovac
	7	Sputnik V
	-96	Other (Specify)
	8	Has heard of COVID-19 vaccine but does not know any names
	0	None, has not heard of any COVID-19 vaccine
	-99	DO NOT READ: Don't Know
	-97	DO NOT READ: Refused
Other, specify:		
E4. Have you received the COVID-19 vaccine?<br>*Read all options aloud*	1	Yes, full dose
	2	Yes, incomplete dose
	0	No
	-97	DO NOT READ: Refused
E5. Which vaccine did you receive?<br>*Do not read options aloud. Select all that apply.*	1	AstraZeneca
	2	Pfizer-BioNTEch
	3	Moderna
	4	Johnson and Johnson
	5	Sinopharm
	6	Sinovac
	7	Sputnik V
	-96	Other (Specify)
	8	Has heard of COVID-19 vaccine but does not know any names
	0	None, has not heard of any COVID-19 vaccine
	-99	DO NOT READ: Don't Know
	-97	DO NOT READ: Refused
Other, specify:		

Question	Answer	
E6. Why did you receive the COVID-19 vaccine?<br>*Do not read options aloud. Select all that apply.*	1	To protect self / others from COVID-19
	2	High perceived risk of getting COVID-19
	3	Travel purposes
	4	Recommendation from health workers
	5	Prioritized due to occupation
	6	Prioritized due to health
	-96	Other (Specify)
	-99	DO NOT READ: Don't Know
	-97	DO NOT READ: Refused
Other, specify:		
E7. Did you experience any side effects within 7 days following the first dose?	1	Yes
	0	No
	-97	DO NOT READ: Refused
E8. Which side effects did you experience following the first dose?<br>*Do not read options aloud. Select all that apply.*	1	Fever
	2	Fatigue
	3	Headache
	4	Muscle soreness/pain (myalgia)
	5	Allergic reaction
	6	Injection site reaction
	-96	Other (Specify)
	-97	DO NOT READ: Refused
Other, specify:		
E9. Did you experience any side effects within 7 days following the second dose?	1	Yes
	0	No
	-97	DO NOT READ: Refused
E10. Which side effects did you experience following the second dose?<br>*Do not read options aloud. Select all that apply.*	1	Fever
	2	Fatigue
	3	Headache
	4	Muscle soreness/pain (myalgia)
	5	Allergic reaction
	6	Injection site reaction
	-96	Other (Specify)
	-97	DO NOT READ: Refused
Other, specify:		

Question	Answer	
E11. Why haven't you ever received the COVID-19 vaccine?<br>*Do not read options aloud. Select all that apply.*	1	Safety concerns / fear of adverse events
	2	Doubt vaccine effectiveness
	3	Do not fear COVID-19 / trust immunity
	4	Don't know where to access vaccine from
	5	Religious beliefs
	6	COVID-19 is a hoax / politics
	7	Not among eligible group
	8	Not having time
	9	Transport costs
	10	Cost of vaccine
	11	Vaccines are unavailable
	-96	Other (Specify)
	-99	DO NOT READ: Don't know
	-97	DO NOT READ: Refused
Other, specify:		
E12. Do you intend to receive the COVID-19 vaccine if it is available?	1	Yes
	0	No
	-97	DO NOT READ: Refused
E13. Why do you intend to receive the COVID-19 vaccine?<br>*Do not read options aloud. Select all that apply.*	1	To protect self / others from COVID-19
	2	High perceived risk of getting COVID-19
	3	Travel purposes
	4	Recommendation from health workers
	5	Prioritized due to occupation
	6	Prioritized due to health
	-96	Other (Specify)
	-99	DO NOT READ: Don't Know
	-97	DO NOT READ: Refused
Other, specify:		
E14. Why don't you intend to receive the COVID-19 vaccine?<br>*Do not read options aloud. Select all that apply.*	1	Safety concerns / fear of adverse events
	2	Doubt vaccine effectiveness

Question	Answer	
	3	Do not fear COVID-19 / trust immunity
	4	Don't know where to access vaccine from
	5	Religious beliefs
	6	COVID-19 is a hoax / politics
	7	Not among eligible group
	8	Not having time
	9	Transport costs
	10	Cost of vaccine
	11	Vaccines are unavailable
	-96	Other (Specify)
	-99	DO NOT READ: Don't know
	-97	DO NOT READ: Refused
Other, specify:		
E15. Has anyone in your household received their COVID-19 vaccine?	1	Yes, specify
	0	No
	-99	DO NOT READ: Don't Know
	-97	DO NOT READ: Refused
E16. In your household, who has received their COVID-19 vaccine? *Do not read options aloud. Select all that apply.*	1	Parents
	2	Children
	3	Grandparents
	4	Grandchildren
	5	Siblings
	6	Cousins
	7	Aunts and Uncles
	8	Spouse
	-96	Other (Specify)
	-97	DO NOT READ: Refused
Other, specify:		
E17. Do you know anyone personally in your community who has received their COVID-19 vaccine?	1	Yes, specify
	0	No
	-99	DO NOT READ: Don't Know
	-97	DO NOT READ: Refused

Question	Answer	
E18. Among those you know personally in your community, who has received their COVID-19 vaccine?<br>*Do not read options aloud. Select all that apply.*	1	Traditional leaders
	2	Religious leaders
	3	Colleagues
	4	Personal friends
	5	Neighbors
	-96	Other (Specify)
	-97	DO NOT READ: Refused
Other, specify:		
Next, I am going to ask some questions about COVIDEX.		
E19. Have you ever heard of COVIDEX before?	1	Yes
	0	No
	-97	DO NOT READ: Refused
E20. Has anyone in your household ever bought or used COVIDEX?	1	Yes
	0	No
	-99	DO NOT READ: Don't Know
	-97	DO NOT READ: Refused
E21. Personally, how much trust do you have in COVIDEX?<br>*Read all options aloud*	1	Trust it a great deal
	2	Tend to trust it
	3	Tend to distrust it
	4	Distrust it greatly
	-99	DO NOT READ: Not sure or don't know
	-97	DO NOT READ: Refused
Next, I am going to ask some more questions on COVID-19 vaccines.		
(Randomize order of F2)		
F2. If you were offered the COVID-19 **AstraZeneca** vaccine right now free of cost, would you take the vaccine?<br>*Read all options aloud*	1	Yes
	0	No
	-95	Have not heard of
	-97	DO NOT READ: Refused
F2. If you were offered the COVID-19 **Pfizer-BioNTEch** vaccine right now free of cost, would you take the vaccine?<br>*Read all options aloud*	1	Yes
	0	No
	-95	Have not heard of
	-97	DO NOT READ: Refused
F2. If you were offered the COVID-19 **Moderna** vaccine right now free of cost, would you take the vaccine?<br>*Read all options aloud*	1	Yes
	0	No
	-95	Have not heard of
	-97	DO NOT READ: Refused
F2. If you were offered the COVID-19 **Johnson and Johnson** vaccine right now free of cost, would you take the vaccine?<br>*Read all options aloud*	1	Yes
	0	No
	-95	Have not heard of
	-97	DO NOT READ: Refused

Question	Answer	
F2. If you were offered the COVID-19 **Sinopharm** vaccine right now free of cost, would you take the vaccine?<br>*Read all options aloud*	1	Yes
	0	No
	-95	Have not heard of
	-97	DO NOT READ: Refused
F2. If you were offered the COVID-19 **Sinovac** vaccine right now free of cost, would you take the vaccine?<br>*Read all options aloud*	1	Yes
	0	No
	-95	Have not heard of
	-97	DO NOT READ: Refused
F2. If you were offered the COVID-19 **Sputnik V** vaccine right now free of cost, would you take the vaccine?<br>*Read all options aloud*	1	Yes
	0	No
	-95	Have not heard of
	-97	DO NOT READ: Refused
F3. Why would you choose these vaccines?	1	More effective
	2	Less adverse events
	3	Used in Western countries
	4	Trust in source
	-96	Other (Specify)
	-99	DO NOT READ: Don't Know
	-97	DO NOT READ: Refused
Other, specify		
F4. If you needed information on vaccines, who in your community would you talk to for advice?		
F5. What are your sources of information on COVID-19?<br>*Do not read options aloud. Select all that apply.*	1	Family members
	2	Friends/peers
	3	Health worker
	4	Phone (messages and calls)
	5	Radio
	6	Television
	7	Church/Mosque
	8	Community member/village health team member
	9	Local leader
	10	Social media (Facebook, WhatsApp, Twitter)
	11	Internet
	-96	Other (Specify)
	-99	DO NOT READ: Don't Know
	-97	DO NOT READ: Refused

Question		Answer
Other, specify		
F6. Which three sources of information do you trust the most? *Do not read options aloud. Select all that apply.*	1	Family members
	2	Friends/peers
	3	Health worker
	4	Phone (messages and calls)
	5	Radio
	6	Television
	7	Church/Mosque
	8	Community member/village health team member
	9	Local leader
	10	Social media (Facebook, WhatsApp, Twitter)
	11	Internet
	-96	Other (Specify)
	-99	DO NOT READ: Don't Know
	-97	DO NOT READ: Refused
Other, specify		
F7. Have you ever been tested at a health facility or laboratory and found to have COVID-19?	1	Yes
	0	No
	-97	DO NOT READ: Refused
F8. Was this COVID infection before or after you received the full dose of the vaccine?	1	Before
	2	After
	3	Both
	-97	DO NOT READ: Refused
B1. What is your highest level of education?	1	No school
	2	Some primary
	3	Complete primary
	4	Secondary - ordinary
	5	Secondary - advanced
	6	Tertiary
	-96	Other (Specify)
	-97	DO NOT READ: Refused
Other, specify		
B2. What is your religion?	1	Catholic
	2	Anglican
	3	Born Again/Pentecostal
	4	Muslim
	-96	Other (Specify)
	-97	DO NOT READ: Refused

Question	Answer	
Other, specify		
B3. On average, how much money do you earn per month?<br>*In Ugandan Shillings*	1	under 50,000
	2	50,001 - 100,000
	3	100,001 - 200,000
	4	200,001 - 500,000
	5	500-001 - 1,000,000
	6	1,000,001 and above
	-97	DO NOT READ: Refused
B4. Does your household have **a television**?	1	Yes
	0	No
	-99	DO NOT READ: Don't Know
	-97	DO NOT READ: Refused
B4. Does your household have **electricity**?	1	Yes
	0	No
	-99	DO NOT READ: Don't Know
	-97	DO NOT READ: Refused
B4. Does your household have **a computer**?	1	Yes
	0	No
	-99	DO NOT READ: Don't Know
	-97	DO NOT READ: Refused
B4. Does your household have a **sofa set**?	1	Yes
	0	No
	-99	DO NOT READ: Don't Know
	-97	DO NOT READ: Refused
B4. Does your household have a **refrigerator**?	1	Yes
	0	No
	-99	DO NOT READ: Don't Know
	-97	DO NOT READ: Refused
B5. Does anyone in your household own a **cassette/CD/DVD player**?	1	Yes
	0	No
	-99	DO NOT READ: Don't Know
	-97	DO NOT READ: Refused

B6A. What is your current occupation?<br>*Read all options aloud*	1	Unemployed/retiree/housewife
	2	Employed
	3	Self-employed
	4	Casual laborer
	5	Farmer
	-96	Other (Specify)
	-97	DO NOT READ: Refused
Other, specify:		
B7A. How many people stay in your home, currently?		
