# Supplementary material for: Uptake of COVID-19 vaccines and associated factors among adults in Uganda: a cross-sectional survey
Source: BMJ Open. 2023 Mar 17;13(3):e067377. doi: 10.1136/bmjopen-2022-067377 (PMC10030279; doi:10.1136/bmjopen-2022-067377)
Supplement: Author's manuscript [file bmjopen-2022-067377.draft_revisions.pdf]

BMJ Open

BMJ Open is committed to open peer review. As part of this commitment we make the peer review history of every article we publish publicly available.

When an article is published we post the peer reviewers' comments and the authors' responses online. We also post the versions of the paper that were used during peer review. These are the versions that the peer review comments apply to.

The versions of the paper that follow are the versions that were submitted during the peer review process. They are not the versions of record or the final published versions. They should not be cited or distributed as the published version of this manuscript.

BMJ Open is an open access journal and the full, final, typeset and author-corrected version of record of the manuscript is available on our site with no access controls, subscription charges or pay-per-view fees (http://bmjopen.bmj.com).

If you have any questions on BMJ Open's open peer review process please email info.bmjopen@bmj.com

Uptake of COVID-19 vaccines and associated factors among adults in Uganda: a cross-sectional survey

Journal:	*BMJ Open*
Manuscript ID	bmjopen-2022-067377
Article Type:	Original research
Date Submitted by the Author:	10-Aug-2022

Complete List of Authors:	Ndejjo, Rawlance; Makerere University, Department of Disease Control and Environmental Health, School of Public Health, College of Health Sciences<br>Chen, Nuole; Massachusetts Institute of Technology<br>Kabwama , Steven ; Makerere University, Department of Community Health and Behavioral Sciences, School of Public Health, College of Health Sciences<br>Namale, Alice; Makerere University, Department of Disease Control and Environmental Health, School of Public Health, College of Health Sciences<br>Wafula, Solomon; Makerere University, Department of Disease Control and Environmental Health, School of Public Health, College of Health Sciences<br>Wanyana, Irene; Makerere University, Department of Epidemiology and Biostatistics, School of Public Health, College of Health Sciences<br>Kizito, Susan; Makerere University, Department of Disease Control and Environmental Health, School of Public Health, College of Health Sciences<br>Kiwanuka, Suzanne ; Makerere University, Department of Health Policy, Planning and Management, School of Public Health, College of Health Sciences<br>Sambisa, William; Bill & Melinda Gates Foundation<br>Tsai, Lily ; Massachusetts Institute of Technology<br>Wanyenze, Rhoda K; Makerere University, 1. Department of Disease Control and Environmental Health, School of Public Health, College of Health Sciences

Keywords:	COVID-19, PUBLIC HEALTH, INFECTIOUS DISEASES

SCHOLARONE™ Manuscripts

I, the Submitting Author has the right to grant and does grant on behalf of all authors of the Work (as defined in the below author licence), an exclusive licence and/or a non-exclusive licence for contributions from authors who are: i) UK Crown employees; ii) where BMJ has agreed a CC-BY licence shall apply, and/or iii) in accordance with the terms applicable for US Federal Government officers or employees acting as part of their official duties; on a worldwide, perpetual, irrevocable, royalty-free basis to BMJ Publishing Group Ltd ("BMJ") its licensees and where the relevant Journal is co-owned by BMJ to the co-owners of the Journal, to publish the Work in this journal and any other BMJ products and to exploit all rights, as set out in our licence.

The Submitting Author accepts and understands that any supply made under these terms is made by BMJ to the Submitting Author unless you are acting as an employee on behalf of your employer or a postgraduate student of an affiliated institution which is paying any applicable article publishing charge ("APC") for Open Access articles. Where the Submitting Author wishes to make the Work available on an Open Access basis (and intends to pay the relevant APC), the terms of reuse of such Open Access shall be governed by a Creative Commons licence – details of these licences and which Creative Commons licence will apply to this Work are set out in our licence referred to above.

Other than as permitted in any relevant BMJ Author's Self Archiving Policies, I confirm this Work has not been accepted for publication elsewhere, is not being considered for publication elsewhere and does not duplicate material already published. I confirm all authors consent to publication of this Work and authorise the granting of this licence.

For peer review only

Uptake of COVID-19 vaccines and associated factors among adults in Uganda: a cross-sectional survey

Rawlance Ndejjo[1]*, Nuole Chen[2], Steven N. Kabwama[3], Alice Namale[1], Solomon T. Wafula[1], Irene Wanyana[4], Susan Kizito[1], Suzanne Kiwanuka[5], William Sambisa[6], Lily L. Tsai[2], Rhoda K. Wanyenze[1]

Affiliations

1. Department of Disease Control and Environmental Health, School of Public Health, College of Health Sciences, Makerere University, Kampala, Uganda
2. Massachusetts Institute of Technology, Massachusetts Avenue, Cambridge, USA
3. Department of Community Health and Behavioral Sciences, Makerere University School of Public Health, Kampala, Uganda
4. Department of Epidemiology and Biostatistics, School of Public Health, College of Health Sciences, Makerere University, Kampala, Uganda
5. Department of Health Policy, Planning and Management, School of Public Health, College of Health Sciences, Makerere University, Kampala, Uganda
6. Bill & Melinda Gates Foundation, Seattle, Washington, USA

* Correspondence, Rawlance Ndejjo

Department of Disease Control and Environmental Health, School of Public Health, College of Health Sciences, Makerere University, Kampala, Uganda, P.O Box 7072, Kampala, Uganda

Email: rndejjo@musph.ac.ug

For peer review only

For peer review only - http://bmjopen.bmj.com/site/about/guidelines.xhtml

Abstract

Objective The Coronavirus Disease (COVID-19) pandemic remains one of the most significant public health challenges ever faced globally. Vaccines are key to ending the pandemic as well as minimize its consequences. This study assessed the uptake of COVID-19 vaccines and associated factors among adults in Uganda.

Design, setting and participants We conducted a cross-sectional mobile phone survey among 1173 adults across the four regions of Uganda.

Main outcome variable Participants reported their uptake of COVID-19 vaccines.

Results Overall, 49.7% had received COVID-19 vaccines with 19.2% having obtained a full dose and 30.5% an incomplete dose. Among the unvaccinated, 91.0% indicated intention to vaccinate. Major reasons for vaccine uptake were protection of self from COVID-19 (86.8%) and a high perceived risk of getting the virus (19.6%). On the other hand, non-uptake was related to vaccine unavailability (42.4%), lack of time (24.1%), and perceived safety (12.5%) and effectiveness concerns (6.9%). The factors associated with receiving the COVID-19 vaccines were older age (≥ 65 years) (APR = 1.32 (95% CI: 1.08 – 1.61), secondary (APR = 1.36 (95% CI: 1.12 – 1.65), or tertiary education (APR = 1.62 (95% CI: 1.31 – 2.00), and health workers as a source of information on COVID-19 (APR = 1.26 (95% CI: 1.10 – 1.45). Also, those who reported a medium-income (APR = 1.24 (1.02 – 1.52) and those resident in Northern (APR = 1.55, 95%CI 1.18 – 2.02) and Central regions (APR = 1.48, 95%CI 1.16 – 1.89) had a higher uptake of COVID-19 vaccines.

Conclusions Uptake of COVID-19 vaccines was moderate in this sample and was associated with older age, secondary and tertiary education, medium-income, region of residence, and

For peer review only

For peer review only - http://bmjopen.bmj.com/site/about/guidelines.xhtml

health workers as a source of COVID-19 information. Efforts are needed to increase access to vaccines and should utilize health workers as champions to enhance uptake.

Wordcount: 3250

Keywords: associated factors, COVID-19, intention, uptake, Uganda, vaccines, willingness

STRENGTHS AND LIMITATIONS OF THIS STUDY

- This study had a high response rate with over 94% of the participants consenting to participate in the phone survey.
- Results from the backchecking with the same individuals showed high consistency with the survey results.
- Being a mobile phone survey, the study participants were not representative of the population as only those with a mobile phone could participate.
- Reporting of vaccination status could have been subject to social desirability bias, which we minimized by reminding participants that the study was only for research purposes.

For peer review only - http://bmjopen.bmj.com/site/about/guidelines.xhtml

INTRODUCTION

The Coronavirus disease of 2019 (COVID-19) has resulted in significant morbidity and mortality globally and negatively disrupted multiple socio-economic sectors. As of 31st March 2022, over 488 million confirmed cases and 6.1 million deaths had been registered globally [1]. In Africa, more than 11 million confirmed COVID-19 cases and 251,953 deaths were reported since the onset of the epidemic. Within the same period, Uganda recorded 163,905 cumulative COVID-19 cases and 3,596 confirmed deaths [1]. In response and under the advice of the World Health Organization, many countries at the beginning of the pandemic implemented non-pharmaceutical interventions (NPIs) that restricted movement such as lockdowns and curfews. Several governments both globally and in Africa also closed schools, places of worship, recreation centres, and public places. Governments also promoted regular hand and respiratory hygiene, wearing of facemasks, ensuring physical and social distancing, and working from home [2]. These public health and social measures significantly impacted the delivery of routine health care services, caused job losses, disrupted education and formal and informal trade, and increased gender-based violence and mental health disorders [3-5].

Vaccines as key pharmaceutical interventions to contain COVID-19 were adopted almost one year into the pandemic globally. Uganda recorded its first confirmed case of COVID-19 on 21st March 2020 and received its first batch of COVID-19 vaccines one-year later in March 2021. At the start, vaccination targeted high-risk groups including health workers, teachers, security personnel, persons older than 50 years, and those with co-morbidities. Starting August 2021 when the country received more doses of vaccines, vaccination was opened up to all Ugandans aged 18 years and above. Vaccines were largely available through designated health facilities, outreaches and mobile vaccination service points. The Ministry of Health (MoH) ran media

For peer review only - http://bmjopen.bmj.com/site/about/guidelines.xhtml

campaigns to mobilise communities for COVID-19 vaccination working hand in hand with local government structures. High vaccination coverage was critical for containment of the pandemic, re-opening of the economy and reversal of the negative socio-economic impacts of the NPIs. However, the opening up of eligibility for vaccination was marred with negative information and fears of vaccine hesitancy. In order to develop critical strategies to achieve high vaccination coverage, there is need for an in-depth understanding of factors influencing the uptake of COVID-19 vaccination. This study, therefore, sought to gather and analyse data to determine the uptake of COVID-19 vaccines and associated factors among adults in Uganda.

METHODS

Study setting

This study was conducted in Uganda located in Eastern Africa. The country has 136 districts distributed in four administrative regions (Northern, Eastern, Central, and Western) which were all involved in the study. As of 2020, Uganda had an estimated population of approximately 41.8 million people [6]. Having registered its first confirmed case of COVID-19 in March 2020, the country had by November 2021 experienced two waves of the disease. The first wave of the pandemic occurred from August 2020 to February 2021 of various non-Delta variants while the second wave happened from May to October 2021 fueled by the Delta variant [1 7].

Study design and population

This was a cross-sectional mobile phone survey conducted in November 2021 among a nationally constituted sample of adults. The study enrolled persons aged ≥18 years sampled from the country's four administrative regions: Central, Eastern, Northern and Western. We excluded persons who said they were ill and unable to participate in the interview.

For peer review only - http://bmjopen.bmj.com/site/about/guidelines.xhtml

Sample size estimation

To enable tracking changes in adherence to NPIs following the introduction of vaccines, we used a previous sample of study respondents from an earlier survey [8] whose data were collected in March 2021 [8]. The sample size for the previous survey was determined using the Leslie Kish formula for cross-sectional studies [9] considering the following assumptions: Two-sided Z statistic corresponding to a 95% confidence interval (1.96), NPI adherence level of 50% since no other study had been conducted to show the composite level of adherence, a precision of 5% and a design effect of 2.5 [8]. Considering a non-response rate of 10%, the total sample size estimate was 1056 people.

Sampling strategy

We used the sample from an earlier survey [8], which was constituted following quota sampling. Quotas were set on age, gender and location proportionate to national COVID-19 case distribution statistics as of February 2021 [10]. With quotas in place, a simple randomly selected (SRS) sample was obtained among the eligible population using a database of phone contacts of previous survey respondents provided by a registered research firm. In cases of replacement of previous participants due to unavailability or refusal to participate, a similar case distribution was followed during sampling of new contacts.

Data collection

Data were collected using a structured survey questionnaire, with mostly closed-ended questions, informed by a review of published literature [8 11 12]. The questionnaire was pretested among 20 people from the four regions of Uganda and relevant adjustments were made. The questionnaire was translated into nine major local languages spoken in Uganda, namely: *Ateso, Luganda,*

For peer review only - http://bmjopen.bmj.com/site/about/guidelines.xhtml

Lugbara, Lugisu, Luo, Lusoga, Ngakarimojong, Runyankole-Rukiga and *Runyoro-Rutooro.* A separate group of translators validated the questionnaire translations and any discrepancies were addressed. The final survey instrument in each language was programmed in SurveyCTO software, incorporating appropriate routing, conditional logic, and other controls and uploaded on hand-held mobile tablets. Bench testing of the survey questionnaire was conducted, and adjustments made before actual data collection. Trained research assistants with a minimum of a Diploma in a health-related field, fluent in the survey languages and with experience in mobile surveys conducted the interviews. Research assistants made phone calls from a designated place in Kampala to the respondents from whom they sought verbal informed consent after explaining to them what the study entailed and entered data into the tablets. Respondents who preferred to defer the phone interviews due to busy schedules or other reasons received follow-up phone calls based on agreed-upon appointment times. Daily checks of the survey data were conducted to monitor quality and intervene early and appropriately, as well as ensure adherence to established quotas. A team of supervisors oversaw the work of the research assistants ensuring that questions were asked appropriately, and respondents were interviewed in the language they were most comfortable with. At the end of the interview period, we conducted back checking of 10% of respondents to ascertain the quality of collected data.

Data management and analysis strategy

During data collection, each research assistant examined, edited, and cleaned their data daily before uploading it to the server. Data were encrypted and anonymized on the server and later downloaded and exported to Stata 15.0 for further cleaning. Data analysis was conducted in Rstudio Version 1.4.1106 (RStudio, PBC). Descriptive statistics have been provided in the form of means (standard deviation) for continuous variables while categorical variables have been

For peer review only - http://bmjopen.bmj.com/site/about/guidelines.xhtml

expressed as frequencies and percentages. Socioeconomic status was generated as an additive index from 6 variables on household ownership of television, computer, sofa set, refrigerator, and cassette/CD/DVD player, and access to electricity. The socio-economic status index was then divided into tertiles. The dependent variable was self-reported uptake of COVID-19 vaccines, which constituted those who reported receiving at least one dose of any World Health Organization (WHO) approved COVID-19 vaccines. We also determined the intention to uptake COVID-19 vaccines by asking unvaccinated respondents if they intended to receive the vaccine. The independent variables included socio-demographic characteristics (age, gender, employment status, education and occupation, place of residence (urban vs. rural, region) and source of information on COVID-19. To determine the factors associated with vaccination uptake, we ran multivariable modified Poisson regressions with robust error variance and presented prevalence ratios and corresponding 95% confidence intervals. Only variables with a p-value ≤ 0.2 at bivariate levels were included in the final model.

Patient and public involvement

No patients or members of the public were involved in the study design, setting the research questions, interpretation or writing up of results, or reporting of the research.

For peer review only

For peer review only - http://bmjopen.bmj.com/site/about/guidelines.xhtml

RESULTS

Sociodemographic characteristics of participants

Of the 1,249 respondents reached, a total of 1,173 (94%) participants completed the survey. The mean age of respondents was 39.7 years (SD ±14.2) and majority 717 (61.1%) were males. Half 606 (51.7%) of the study participants were from the Central region, 548 (46.8%) had an urban residence and 548 (46.7%) belonged to the lowest socioeconomic tertile. Nearly four in ten (39%) respondents had only primary or no formal education (Table 1).

Table 1: Sociodemographic characteristics of participants

Characteristic	Number of participants (N = 1,173)	Percentage (%)[1]
**Sex**		
Male	717	61.1
Female	456	38.9
**Age group (years), Mean age (SD)**	39.7 (±14.2)	
18 – 35	553	47.1
36 – 55	439	37.4
56 - 64	92	7.8
65+	89	7.6
**Region of residence**		
North	182	15.5
East	211	18
Central	606	51.7
West	174	14.8
**Residence**		
Urban	548	46.8
Rural	417	35.6
Semi-urban	207	17.7
Not stated	1	
**Earnings per month ($)**		
< 14	256	25.6
14 – 29	226	22.6
30 – 57	196	19.6
58 – 143	229	22.9
> 143	93	9.3

For peer review only

For peer review only - http://bmjopen.bmj.com/site/about/guidelines.xhtml

Characteristic	Number of participants (N = 1,173)	Percentage (%)[1]
Not stated	173	
**Education level**		
None or Incomplete Primary	265	23.2
Primary (completed)	180	15.7
Secondary	431	37.7
Tertiary	268	23.4
Not stated	27	
**Socio-economic index**		
Low	548	46.7
Middle	435	37.1
Higher	190	16.2
**Religion**		
Catholic	384	33.3
Anglican	372	32.3
Born Again (Pentecostal)	147	12.8
Muslim	226	19.6
Other religions	24	2.1
Not stated	20	
**Current Occupation**		
Unemployed	193	17.1
Employed	182	16.1
Self employed	355	31.4
Casual laborer	67	5.9
Farmer	334	29.5
Not stated	42	
**Current household size, Mean (SD)**	5.6 (3.5)	
5 or fewer	653	55.7
6 – 10	430	36.7
More than 10	90	7.7

[1] *Percentages calculated do not include respondents who did not record responses (e.g. "Not stated" in the tables)*

Uptake of COVID-19 vaccines and intention to vaccinate

Among all respondents, 225 (19.2%) reported receiving a full dose of the vaccine and 357 (30.5%) an incomplete dose. Slightly above sixty per cent of the respondents 367 (63.2%) reportedly experienced side effects following vaccination mostly fever 147 (40.1%), fatigue 115

For peer review only - http://bmjopen.bmj.com/site/about/guidelines.xhtml

(31.3%) and headache 101 (27.5%). Among those who had not received a vaccine, 537 (91.8%) reported intention to vaccinate (

Table 2).

Table 2: Vaccination uptake and intention to vaccinate among participants

Variable	Count	Percentage (%)[1]
**Vaccination uptake**	**(n = 582)**	
Full dose (two shots)	225	19.2
Incomplete dose	357	30.5
No vaccination	590	50.3
**Experienced any side effects after first dose**		
No	214	36.8
Yes	367	63.2
**Side effects reported**	**(n = 367)**	
Fever	147	40.1
Fatigue	115	31.3
Headache	101	27.5
Muscle soreness /pain	95	25.9
Injection site reaction	88	24.0
Others[2]	38	10.4
**Vaccination intention (among unvaccinated)**	**n = 590**	
Intend to vaccinate	537	91.0
Did not intend to vaccinate	48	8.1
Did not know	5	0.8

[1] Percentages calculated do not include respondents who did not record responses e.g. "Not stated" in the tables
[2] Allergic reaction, cough, body pain, dizziness, arrhythmias, body weakness, paralysis for a few days, erectile dysfunction for a few days

For peer review only - http://bmjopen.bmj.com/site/about/guidelines.xhtml

Reasons for vaccine uptake/ non-uptake and intention/un intention to vaccinate

The reasons for COVID-19 vaccine uptake and intention to vaccinate were similar with both categories of respondents mostly reporting the need to obtain protection from COVID-19 and having a high perceived risk of getting the virus. Over 40% of respondents who had not been vaccinated attributed it to vaccine unavailability 250 (42.4%) and below a quarter of respondents to not having time 142 (24.1%). The reasons for lack of intention to vaccinate were mainly related to safety 24 (50.0%) and effectiveness concerns 17 (35.4%) which were similarly reported for non-uptake of vaccines (

For peer review only

For peer review only - http://bmjopen.bmj.com/site/about/guidelines.xhtml

Table 3).

Table 3: Reasons for (non) uptake of COVID-19 vaccines and intention to vaccinate (multiple response)

Reasons	Uptake of vaccines n = 582 (%)	Intention to vaccinate n = 537 (%)
To protect self from COVID-19	505 (86.8%)	458 (85.3%)
High perceived risk of getting COVID-19	114 (19.6)	90 (16.8%)
Prioritized due to health (comorbidities)	95 (16.3)	34 (6.3%)
Recommendation from health workers	81 (13.9)	38 (7.1)
Prioritized due to occupation	74 (12.7)	-
Travel purposes	44 (7.6)	45 (8.4%)

For peer review only - http://bmjopen.bmj.com/site/about/guidelines.xhtml

For peer review only

Job requirement	-	82 (15.3%)
Others	21 (3.6)[1]	20 (3.8)[2]
**Reasons**	**Non uptake of vaccines n = 590 (%)**	**No intention to vaccinate n = 48 (%)**
Vaccines are unavailable	250 (42.4)	1 (2.1)
Don’t have time	142 (24.1)	2 (4.2)
Safety concerns	74 (12.5)	24 (50.0)
Doubt vaccine effectiveness	41 (6.9)	17 (35.4)
Not among eligible group	30 (5.1)	4 (8.3)
Transport costs	24 (4.1)	Not reported
Don’t know where to access the vaccines from	20 (3.4)	Not reported
Do not fear COVID-19/ trust immunity	10 (1.7)	1 (2.1)
Others	82 (14.0)[3]	7 (14.6)[4]

[1] Requirement for school attendance, being exemplary, following MOH guidelines, boosting immunity, to access services, among the eligible group.

[2] Access to health services, government mandate, pressure from peers, to be exemplary, requirement for school attendance.

[3] Pregnant, breastfeeding, waiting for another vaccine type, lack identification documents, long queues, currently sick, recently recovered from COVID-19.

[4] Religious beliefs, do not believe COVID-19, HIV positive and fear side effects, underlying Hepatitis B infection, Body already weak, Lack of identification documents.

Willingness to vaccinate for different vaccine types

All respondents were asked if they would receive the different types of COVID-19 vaccines if offered at that point and were free of charge. Only 316 (26.9%) reported that they would take any vaccine regardless of the type and 488 (41.6%) indicated a willingness to take at least one type of the vaccine. The most preferred COVID-19 vaccines types were Johnson and Johnson 436 (37.4%) and AstraZeneca 405 (34.7%) (Figure 1).

Figure 1 Willingness for COVID-19 vaccination for different vaccine types

For peer review only

For peer review only - http://bmjopen.bmj.com/site/about/guidelines.xhtml

Factors associated with uptake of COVID-19 vaccines

At the multivariable analysis level, participants aged ≥65 years had a 32% higher likelihood to have been vaccinated compared to those aged 18-35 years (Adjusted PR = 1.32, 95% CI 1.08 – 1.61, p = 0.008). Participants from the Northern (adjusted PR = 1.55, 95% CI 1.18 – 2.02, p = 0.002) and Central regions (adjusted PR = 1.48, 95% CI 1.16 – 1.89, p = 0.002) respectively had a 55% and 48% higher likelihood to have received the vaccine compared to those from the Western region. Participants with secondary (adjusted PR = 1.36, 95% CI 1.12 – 1.65, p = 0.002) or tertiary education (Adjusted PR = 1.62, 95% CI 1.31 – 2.00, p < 0.001) were more likely to have received the COVID-19 vaccine compared to those with incomplete primary/no formal education. Respondents whose monthly income was between $30 and $57 (APR = 1.24 (1.02 – 1.52), p = 0.029) had a higher uptake of COVID-19 vaccines than those who earned < $14. Having health workers as a source of information on COVID-19 was associated with higher uptake of COVID-19 vaccines in Uganda (adjusted PR = 1.26, 95%CI 1.10 – 1.45, p = 0.001) (Table 4).

Table 4: Factors associated with COVID-19 vaccine uptake among adults

Variables / Characteristics	Self-reported uptake of COVID-19 vaccine		Unadjusted PR (95% CI)[1]	p-value	Adjusted PR (95% CI)[2]	p-value
	No (%)	Yes (%)				
**Age in years**						
18 – 35	296 (53.5)	257 (46.5)	1		1	
36 – 55	213 (48.6)	225 (51.4)	1.11 (0.97 – 1.26)	0.124	1.09 (0.95 – 1.25)	0.244
56 – 64	44 (47.8)	48 (52.2)	1.12 (0.91 – 1.39)	0.292	1.17 (0.92 – 1.48)	0.193
65+	37 (41.6)	52 (58.4)	1.26 (1.03 -1.53)	**0.023**	1.32 (1.08 – 1.61)	**0.008**
**Region of residence**						

For peer review only - http://bmjopen.bmj.com/site/about/guidelines.xhtml

Variables / Characteristics	Self-reported uptake of COVID-19 vaccine		Unadjusted PR (95% CI)[1]	p-value	Adjusted PR (95% CI)[2]	p-value
	No (%)	Yes (%)				
Western	115 (66.5)	58 (33.5)	1		1	
Northern	74 (40.7)	108 (59.3)	1.77 (1.39 – 2.25)	**<0.001**	1.55 (1.18 - 2.02)	**0.002**
Eastern	112 (53.1)	99 (46.9)	1.40 (1.09 – 1.80)	**0.010**	1.29 (0.99 - 1.69)	0.064
Central	289 (47.7)	317 (52.3)	1.56 (1.25– 1.95)	**0.001**	1.48 (1.16 - 1.89)	**0.002**
**Residence**						
Urban	270 (49.4)	277 (50.6)			1	
Rural	206 (49.4)	211 (50.6)	0.99 (0.88 – 1.13)	0.990	1.11 (0.97 – 1.28)	0.137
Semi-urban	114 (55.1)	93 (44.9)	0.89 (0.75 – 1.05)	0.173	0.92 (0.75 – 1.11)	0.373
**Gender**						
Male	351 (49.0)	366 (51.0)	1		1	
Female	239 (52.5)	216 (47.5)	0.93 (0,82 – 1.05)	0.237	1.00 (0.87 – 1.14)	0.973
**Wealth index**						
Low	290 (53.0)	257 (47.0)	1		1	
Middle	217 (49.9)	218 (50.1)	1.07 (0.94 – 1.21)	0.328	1.06 (0.91 – 1.24)	0.442
High	83 (43.7)	107 (56.3)	1.20 (1.03 – 1.40)	**0.021**	1.03 (0.83 – 1.28)	0.758
**Current Occupation**						
Unemployed	91 (47.2)	102 (52.8)	1		1	
Employed	76 (41.8)	106 (58.2)	1.10 (0.92 – 1.32)	0.294	1.03 (0.84 – 1.27)	0.763
Self employed	196 (55.2)	159 (44.8)	0.85 (0.71 – 1.01)	0.066	0.84 (0.68 – 1.02)	0.078
Casual laborer	45 (67.2)	22 (32.8)	0.62 (0.43 – 0.90)	**0.011**	0.73 (0.48 – 1.11)	0.146
Farmer	164 (49.1)	170 (50.9)	0.96 (0.81 – 1.14)	0.664	0.99 (0.82 – 1.19)	0.931
**Education level**						
No formal education / incomplete primary	161 (60.8)	104 (39.2)	1		1	

For peer review only - http://bmjopen.bmj.com/site/about/guidelines.xhtml

Variables / Characteristics	Self-reported uptake of COVID-19 vaccine		Unadjusted PR (95% CI)[1]	p-value	Adjusted PR (95% CI)[2]	p-value
	No (%)	Yes (%)				
Complete primary	109 (60.6)	71 (39.4)	1.01 (9.79 - 1.27)	0.966	1.00 (0.78 – 1.28)	0.998
Secondary education	207 (48.0)	224 (52.0)	1.32 (1.11 – 1.58)	**0.002**	1.36 (1.12 – 1.65)	**0.002**
Tertiary	98 (36.6)	170 (63.4)	1.62 (1.36 – 1.93)	**< 0.001**	1.62 (1.31 – 2.00)	**< 0.001**
**Household size (mean)**	5.41	5.99	1.02 (1.01 – 1.03)	**< 0.001**	1.02 (1.00 – 1.03)	0.071
**Monthly income ($)**						
< 14	144 (56.2)	112 (43.8)	1		1	
14 – 29	117 (51.8)	109 (48.2)	1.10 (0.91 – 1.34)	0.324	1.08 (0.89 – 1.32)	0.423
30 – 57	86 (43.9)	110 (56.1)	1.28 (1.07 – 1.55)	**0.009**	1.24 (1.02 – 1.52)	**0.029**
58 – 143	114 (49.8)	115 (50.2)	1.15 (0.95 – 1.39)	0.154	0.98 (0.79 – 1.22)	0.876
> 143	36 (38.7)	57 (61.3)	1.40 (1.13 – 1.73)	**0.002**	1.16 (0.91 – 1.49)	0.219
**Health workers as source of information on COVID-19[3]**						
No	245 (57.9)	178 (42.1)	1		1	
Yes	345 (46.1)	404 (53.9)	1.28 (1.13 – 1.45)	**< 0.001**	1.26 (1.10 – 1.45)	**0.001**

[1] *Bivariate analysis*

[2] *Multivariable analysis*

[3]*Other sources of information included family members, friends/peers, Radio, Television, community members and social media among others which were dichotomized and included in the analysis but were not significant*

DISCUSSION

This study examined the uptake of COVID-19 vaccines and associated factors among adults aged 18 years and above in Uganda. Among the study participants, about one in five (19.2%) reported receiving a full dose of the COVID-19 vaccine while 30.5% had received an incomplete dose. Over 90% of those who were unvaccinated reported the intention to be vaccinated. The major reasons for vaccine uptake and intention to vaccinate were protection of self from COVID-

For peer review only

For peer review only - http://bmjopen.bmj.com/site/about/guidelines.xhtml

19 and a high perceived risk of getting the virus while reasons for vaccine non-uptake were vaccine unavailability, the lack of time to go get vaccinated, and safety and effectiveness concerns. The factors that were associated with receiving the COVID-19 vaccine were older age (65 years and above), having secondary education and above, having a moderate income, and reporting health workers as a source of information on COVID-19. Being a resident of Northern and Central Uganda was also associated with a high likelihood of receiving the vaccine.

Uptake of COVID-19 vaccines in this sample of respondents was higher for both full and incomplete doses than the vaccinated proportion of the population as of November 2021 when this study was conducted. Ministry of Health data of 8th November 2021 indicated that 55.8% and 16.8% of the priority groups and 12.2% and 3.7% of the adult population had received their first and second doses of the vaccine respectively [13]. The higher-than-baseline vaccination coverage could be attributed to the use of mobile phones for the survey and thus the relatively urbanized study sample whose access to vaccines was higher than those in rural areas. Moreover, a high proportion of participants were from the Central region, which was most impacted by COVID-19, and their experiences could have influenced vaccine uptake. In addition, intention to vaccinate was very high at over 90%; higher than the combined "definite intention" of 57.8% and "probable intention" of 26.2% from the March survey round [8]. In a November 2021 survey among 23,000 respondents from 19 African Union members states including Uganda, (78%) of respondents had either been vaccinated or were likely to get vaccinated [14]. The second COVID-19 wave fueled by the Delta variant that was experienced in Uganda from June to September 2021 and led to at least 2,800 deaths compared to the less than 300 recorded at the end of the first wave [1 15] could also have contributed to the high uptake of the vaccine and intention-to-vaccinate. In addition, there was concern about potential vaccine mandates including anticipation

For peer review only - http://bmjopen.bmj.com/site/about/guidelines.xhtml

that the unvaccinated would be denied health and social services which could also have increased the intention to vaccinate.

The major reasons for vaccine uptake and intention to vaccinate were protection of self from COVID-19 and a high perceived risk of getting the virus, similar to previous research [11]. This is also an indication of the respondents' appreciation of the role of vaccines in preventing morbidity and saving lives. Those unvaccinated attributed it to vaccine unavailability and the lack of time. The survey in 19 African countries concluded that low vaccine uptake was mostly due to unpredictable supply of vaccines and logistical hurdles than reluctance or refusal to get vaccinated [14]. To bridge the willingness-intention-uptake gap in Uganda, the Ministry of Health should increase access and availability of COVID-19 vaccines. Evidence shows that strategies that take vaccines closer to the communities are likely to mitigate time and transport-related barriers and increase vaccine uptake [16 17]. This could be achieved by increasing the number of health facilities offering the vaccines, conducting more vaccination outreaches, or setting up mobile vaccine points. The World Health Organization guidance has also emphasized the importance of location and time in COVID-19 vaccine uptake [18]. On the other hand, the study reported that safety and effectiveness concerns hindered vaccine uptake and intention to vaccinate similar to previous research [8 11 19-22]. Of note as well was the observed high prevalence (63%) of self-reported vaccine side effects which could go a long way in reinforcing safety concerns among the population. Vaccine adverse events should be monitored closely, and appropriate information, education and communication material developed including information on expected side effects to counter their potential effect on the uptake of vaccination by the unvaccinated. Accurate, consistent and transparent communication and dialogue about uncertainty, risks and anticipated benefits can go a long way in building confidence and trust in

For peer review only

For peer review only - http://bmjopen.bmj.com/site/about/guidelines.xhtml

the COVID-19 vaccines and create motivation for vaccination [18 22]. This could also bridge observed gaps in vaccine preference to prevent this from being a barrier to vaccination. The Johnson and Johnson vaccine being a single shot had a higher preference among respondents due to the perceived inconvenience and unpredictability of obtaining a second vaccine dose.

It was not surprising that those aged 65 years and above had a higher vaccination uptake as these were part of the prioritized group for COVID-19 vaccination in the country. Education status also predicted vaccination status similar to previous research on COVID-19 vaccine acceptability [23-26]. However, further efforts are required to ensure the dissemination of accurate and simple COVID-19 vaccination messages to those of lower education levels including translating information in the local languages so that this group is not left behind. A moderate income was associated with higher vaccine uptake; however, this relationship was not sustained with increasing income levels. The regional differences observed in the uptake of COVID-19 vaccines may have been due to differences in vaccine access and availability, especially for Central region which was most hit by the pandemic and was prioritized early during vaccine rollout. From previous research, income levels and locations have been reported as predictors of COVID-19 vaccine acceptability [26 27].

One major finding from our work was that respondents whose source of information on COVID-19 was health workers had a higher likelihood for COVID-19 vaccination. This positions health workers as a key resource in increasing vaccination uptake, and thus they should be furnished with sufficient and accurate information and supported with effective communication tools to influence their clients at facility and community level. Previous studies report that health worker advice on vaccination was most trusted [11 21]. Health workers can lead health education and awareness programs on COVID-19 and use their platforms at health facility and community

For peer review only - http://bmjopen.bmj.com/site/about/guidelines.xhtml

level to influence the masses to uptake COVID-19 vaccines. However, vaccine uptake among health workers themselves was low at the time even when they were prioritized for vaccination from the start of the campaigns in Uganda and elsewhere. In a March 2021 survey in Uganda, just after the launch of the COVID-19 vaccination exercise, a vaccine acceptability rate of 37.3% and hesitancy of 30.7% were reported among medical students [12]. In a June to August 2021 online survey, acceptance or willingness to uptake the COVID-19 vaccine stood at over 97% and 65.3% of eye healthcare workers had received a COVID-19 vaccine shot influenced by high perceived susceptibility and benefits [28]. An in-depth study among health workers reported the lack of trust in the vaccine, fear of side effects, not feeling at risk, lack of sufficient information about vaccines, health systems challenges and religious beliefs as barriers to COVID-19 vaccination [29]. When health workers are vaccinated, they are more likely to recommend the same to their clients [30]. Therefore, appropriate interventions should be instituted to effectively deal with vaccine hesitancy among health workers and have them as champions for COVID-19 vaccination.

Study limitations and strengths

Being a mobile phone survey, the study participants were not representative of the population and only those with a mobile phone could participate, contributing to selection bias. However mobile phone coverage in Uganda has increased over the years; according to the Uganda National Household Survey 2020, 74.0% of Ugandans own mobile phones [31]. There was also potential for social desirability bias, especially regarding reporting vaccination status which we minimized by reminding participants that the study was only for research purposes. Also, as a cross-sectional survey, the direction of associations observed is not clear. On the other hand, our study had a high response rate with over 94% of the participants consenting to participate.

For peer review only - http://bmjopen.bmj.com/site/about/guidelines.xhtml

Results from the backchecking with the same individuals also showed high consistency with the survey results. Our study provides insights into COVID-19 vaccination uptake and intention to vaccinate which can facilitate the development of context-relevant strategies to increase vaccinations.

CONCLUSIONS

Half of the study respondents were vaccinated against COVID-19, which was associated with older age, higher education level, moderate income, region of residence and reporting health workers as the source of COVID-19 information. Among the unvaccinated, over 90% expressed intention to vaccinate. Efforts are needed to increase access to vaccines and utilize health workers as a key resource in sharing information and champions to influence the masses which should positively impact uptake of COVID-19 vaccines.

Acknowledgements

The authors wish to thank the study participants who provided the information that led to this publication. The research assistants are also appreciated for their contribution to this study.

Author Contributions

RN, NC, SNK, AN, WS, LLT and RKW conceptualized and designed the study. RN, NC, SNK, AN, IW, SKiz supported the data collection. RN, NC, SNK, AN, STW, IW, SKiz, SKiw, WS, LLT and RKW contributed to analysis and interpretation of findings. RN, NC, STW wrote the

For peer review only - http://bmjopen.bmj.com/site/about/guidelines.xhtml

first draft of the manuscript. SNK, AN, IW, SKiz, SKiw, WS, LLT and RKW critically reviewed the draft manuscript. All authors read and approved the final manuscript.

Funding

This work was supported in whole or in part, by the Bill & Melinda Gates Foundation [Opportunity ID: INV-019313]. The views, opinions, and content of this publication are those of the authors and do not necessarily reflect the views, opinions, or policies of the Bill and Melinda Gates Foundation.

Competing interests

None declared.

Patient and public involvement

Patients and/or the public were not involved in the design, or conduct, or reporting, or dissemination plans of this research.

Consent for publication

Not applicable

Ethics approval and consent to participate

For peer review only - http://bmjopen.bmj.com/site/about/guidelines.xhtml

Ethical approval to conduct the survey was sought from the Makerere University School of Public Health Higher Degrees Research and Ethics Committee (protocol SPH-2021-150) and the study was registered by the Uganda National Council of Science and Technology (HS1742ES). Verbal consent was provided before participation in the survey. Personally identifiable information including name of respondent, their phone number and the household head name were encrypted with passwords on the SurveyCTO server and drives and was only accessible to the investigator(s). All phones and tablets used for data collection were password-protected to protect respondent data.

Data availability statement

The data are available from the corresponding author on reasonable request.

REFERENCES

1. WHO. COVID-19 Coronavirus Pandemic: World Health Organization; 2021 [Available from: https://www.worldometers.info/coronavirus/#countries accessed 22nd January 2021.

2. WHO. Modes of transmission of virus causing COVID-19: implications for IPC precaution recommendations: scientific brief, 27 March 2020: World Health Organization, 2020.

3. WHO. COVID-19 significantly impacts health services for noncommunicable diseases. 2020 2020 [Available from: https://www.who.int/news-room/detail/01-06-2020-covid-19-significantly-impacts-health-services-for-noncommunicable-diseases.

For peer review only - http://bmjopen.bmj.com/site/about/guidelines.xhtml

4. Vindegaard N, Benros ME. COVID-19 pandemic and mental health consequences: Systematic review of the current evidence. *Brain, behavior, and immunity* 2020;89:531-42.

5. Piquero AR, Jennings WG, Jemison E, et al. Domestic violence during the COVID-19 pandemic - Evidence from a systematic review and meta-analysis. *Journal of Criminal Justice* 2021;74:101806. doi: https://doi.org/10.1016/j.jcrimjus.2021.101806

6. Uganda Bureau of Statistics. National Population Projections by single age and sex (2015–2040). Kampala: Uganda Bureau of statistics; 2020 [accessed 12th August 2021.

7. Elayeete S, Nampeera R, Nsubuga EJ, et al. Comparative epidemiologic analysis of COVID-19 patients during the first and second waves of COVID-19 in Uganda. *IJID Regions* 2022;3:160-67. doi: https://doi.org/10.1016/j.ijregi.2022.03.017

8. Wafula ST, Mugume IB, Sensasi B, et al. Intention to vaccinate against COVID-19 and adherence to non-pharmaceutical interventions against COVID-19 prior to the second wave of the pandemic in Uganda: a cross-sectional study. *BMJ Open* 2022;12(6):e057322. doi: 10.1136/bmjopen-2021-057322 [published Online First: 2022/06/03]

9. Kish L. Survey sampling. Oxford, England: Wiley 1965.

10. Uganda Ministry of Health. National COVID-19 surveillance data, February 2021. : Ministry of Health, 2021.

11. Solís Arce JS, Warren SS, Meriggi NF, et al. COVID-19 vaccine acceptance and hesitancy in low-and middle-income countries. *Nature medicine* 2021;27(8):1385-94.

12. Kanyike AM, Olum R, Kajjimu J, et al. Acceptance of the coronavirus disease-2019 vaccine among medical students in Uganda. *Tropical Medicine and Health* 2021;49(1):37. doi: 10.1186/s41182-021-00331-1

For peer review only - http://bmjopen.bmj.com/site/about/guidelines.xhtml

13. Uganda Ministry of Health. COVID-19 Vaccination Update 10th November 2021. Kampala, Uganda: Ministry of Health, 2021.

14. (PERC) PfE-BRtC-. Responding to COVID-19 in Africa Finding the Balance Part IV, 2021.

15. World Health Organization. Uganda: WHO Coronavirus Disease (COVID-19) Dashboard With Vaccination Data

2022 [Available from: https://covid19.who.int/region/afro/country/ug.

16. Schmid P, Rauber D, Betsch C, et al. Barriers of influenza vaccination intention and behavior–a systematic review of influenza vaccine hesitancy, 2005–2016. *PloS one* 2017;12(1):e0170550.

17. Betsch C, Böhm R, Korn L. Inviting free-riders or appealing to prosocial behavior? game-theoretical reflections on communicating herd immunity in vaccine advocacy. *Health Psychology* 2013;32(9):978.

18. World Health Organization. Behavioural considerations for acceptance and uptake of COVID-19 vaccines: WHO technical advisory group on behavioural insights and sciences for health, meeting report, 15 October 2020. 2020

19. MacDonald NE. Vaccine hesitancy: Definition, scope and determinants. *Vaccine* 2015;33(34):4161-64.

20. Sadique MZ, Devlin N, Edmunds WJ, et al. The effect of perceived risks on the demand for vaccination: results from a discrete choice experiment. *PloS one* 2013;8(2):e54149.

21. Dubé E, Laberge C, Guay M, et al. Vaccine hesitancy: an overview. *Human vaccines & immunotherapeutics* 2013;9(8):1763-73.

22. Betsch C, Böhm R, Chapman GB. Using behavioral insights to increase vaccination policy effectiveness. *Policy Insights from the Behavioral and Brain Sciences* 2015;2(1):61-73.

For peer review only - http://bmjopen.bmj.com/site/about/guidelines.xhtml

23. Echoru I, Ajambo PD, Keirania E, et al. Sociodemographic factors associated with acceptance of COVID-19 vaccine and clinical trials in Uganda: a cross-sectional study in western Uganda. *BMC Public Health* 2021;21(1):1106. doi: 10.1186/s12889-021-11197-7

24. McAbee L, Tapera O, Kanyangarara M. Factors associated with COVID-19 vaccine intentions in Eastern Zimbabwe: A cross-sectional study. *Vaccines* 2021;9(10):1109.

25. Moola S, Gudi N, Nambiar D, et al. A rapid review of evidence on the determinants of and strategies for COVID-19 vaccine acceptance in low-and middle-income countries. *Journal of global health* 2021;11

26. Adebisi YA, Alaran AJ, Bolarinwa OA, et al. When it is available, will we take it? Social media users' perception of hypothetical COVID-19 vaccine in Nigeria. *Pan Afr Med J* 2021;38

27. Abedin M, Islam MA, Rahman FN, et al. Willingness to vaccinate against COVID-19 among Bangladeshi adults: Understanding the strategies to optimize vaccination coverage. *PLoS One* 2021;16(4):e0250495.

28. Otiti-Sengeri J, Andrew OB, Lusobya RC, et al. High COVID-19 Vaccine Acceptance among Eye Healthcare Workers in Uganda. *Vaccines* 2022;10(4):609.

29. Muhamadi L, Edith N, James W, et al. Lack of Trust, Insufficient knowledge and Risk denial; an in-depth Understanding of Health worker Barriers to uptake of the Covid-19 vaccine at Iganga Hospital Eastern Uganda, and Mengo Hospital Kampala Uganda. *medRxiv* 2021

30. Paterson P, Meurice F, Stanberry LR, et al. Vaccine hesitancy and healthcare providers. *Vaccine* 2016;34(52):6700-06.

For peer review only

For peer review only - http://bmjopen.bmj.com/site/about/guidelines.xhtml

31. Uganda Bureau of Statistics (UBOS). Uganda National Household Survey 2019/2020. Kampala, Uganda: UBOS, 2021.

For peer review only

For peer review only - http://bmjopen.bmj.com/site/about/guidelines.xhtml

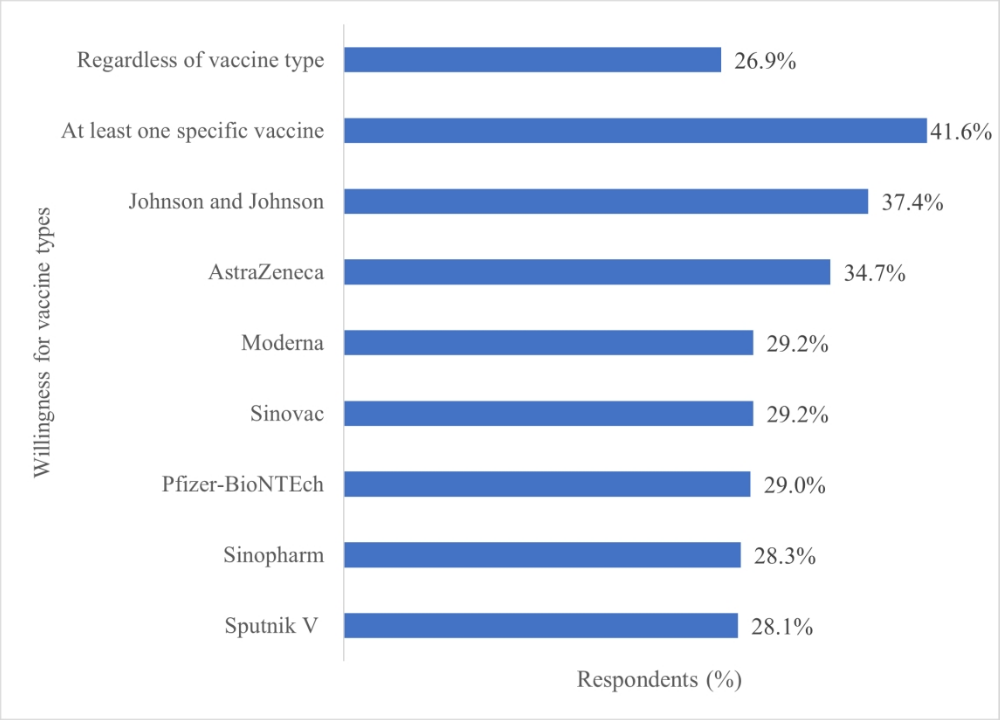

Willingness for COVID-19 vaccination for different vaccine types

408x294mm (130 x 130 DPI)

STROBE Statement—Checklist of items that should be included in reports of ***cross-sectional studies***

	Item No	Recommendation	Page No
**Title and abstract**	1	(*a*) Indicate the study's design with a commonly used term in the title or the abstract	1
		(*b*) Provide in the abstract an informative and balanced summary of what was done and what was found	1
**Introduction**			
Background/rationale	2	Explain the scientific background and rationale for the investigation being reported	4,5
Objectives	3	State specific objectives, including any prespecified hypotheses	5
**Methods**			
Study design	4	Present key elements of study design early in the paper	5
Setting	5	Describe the setting, locations, and relevant dates, including periods of recruitment, exposure, follow-up, and data collection	5
Participants	6	(*a*) Give the eligibility criteria, and the sources and methods of selection of participants	5
Variables	7	Clearly define all outcomes, exposures, predictors, potential confounders, and effect modifiers. Give diagnostic criteria, if applicable	6
Data sources/ measurement	8*	For each variable of interest, give sources of data and details of methods of assessment (measurement). Describe comparability of assessment methods if there is more than one group	6
Bias	9	Describe any efforts to address potential sources of bias	6,20
Study size	10	Explain how the study size was arrived at	6
Quantitative variables	11	Explain how quantitative variables were handled in the analyses. If applicable, describe which groupings were chosen and why	7,8
Statistical methods	12	(*a*) Describe all statistical methods, including those used to control for confounding	7,8
		(*b*) Describe any methods used to examine subgroups and interactions	7,8
		(*c*) Explain how missing data were addressed	7,8
		(*d*) If applicable, describe analytical methods taking account of sampling strategy	Not applicable
		(*e*) Describe any sensitivity analyses	Not applicable
**Results**			
Participants	13*	(a) Report numbers of individuals at each stage of study—eg numbers potentially eligible, examined for eligibility, confirmed eligible, included in the study, completing follow-up, and analysed	9
		(b) Give reasons for non-participation at each stage	9
		(c) Consider use of a flow diagram	Not necessary
Descriptive data	14*	(a) Give characteristics of study participants (eg demographic, clinical, social) and information on exposures and potential confounders	9

For peer review only

For peer review only - http://bmjopen.bmj.com/site/about/guidelines.xhtml

		(b) Indicate number of participants with missing data for each variable of interest	9-16
Outcome data	15*	Report numbers of outcome events or summary measures	9-16
Main results	16	(*a*) Give unadjusted estimates and, if applicable, confounder-adjusted estimates and their precision (eg, 95% confidence interval). Make clear which confounders were adjusted for and why they were included	9-16
		(*b*) Report category boundaries when continuous variables were categorized	9-16
		(*c*) If relevant, consider translating estimates of relative risk into absolute risk for a meaningful time period	Not relevant
Other analyses	17	Report other analyses done—eg analyses of subgroups and interactions, and sensitivity analyses	None
**Discussion**			
Key results	18	Summarise key results with reference to study objectives	17
Limitations	19	Discuss limitations of the study, taking into account sources of potential bias or imprecision. Discuss both direction and magnitude of any potential bias	20,21
Interpretation	20	Give a cautious overall interpretation of results considering objectives, limitations, multiplicity of analyses, results from similar studies, and other relevant evidence	17-21
Generalisability	21	Discuss the generalisability (external validity) of the study results	20,21
**Other information**			
Funding	22	Give the source of funding and the role of the funders for the present study and, if applicable, for the original study on which the present article is based	22

*Give information separately for exposed and unexposed groups.

Note: An Explanation and Elaboration article discusses each checklist item and gives methodological background and published examples of transparent reporting. The STROBE checklist is best used in conjunction with this article (freely available on the Web sites of PLoS Medicine at http://www.plosmedicine.org/, Annals of Internal Medicine at http://www.annals.org/, and Epidemiology at http://www.epidem.com/). Information on the STROBE Initiative is available at www.strobe-statement.org.

For peer review only - http://bmjopen.bmj.com/site/about/guidelines.xhtml

BMJ Open

Uptake of COVID-19 vaccines and associated factors among adults in Uganda: a cross-sectional survey

Journal:	*BMJ Open*
Manuscript ID	bmjopen-2022-067377.R1
Article Type:	Original research
Date Submitted by the Author:	19-Jan-2023
Complete List of Authors:	Ndejjo, Rawlance; Makerere University, Department of Disease Control and Environmental Health, School of Public Health, College of Health Sciences<br>Chen, Nuole; Massachusetts Institute of Technology<br>Kabwama , Steven ; Makerere University, Department of Community Health and Behavioral Sciences, School of Public Health, College of Health Sciences<br>Namale, Alice; Makerere University, Department of Disease Control and Environmental Health, School of Public Health, College of Health Sciences<br>Wafula, Solomon; Makerere University, Department of Disease Control and Environmental Health, School of Public Health, College of Health Sciences<br>Wanyana, Irene; Makerere University, Department of Epidemiology and Biostatistics, School of Public Health, College of Health Sciences<br>Kizito, Susan; Makerere University, Department of Disease Control and Environmental Health, School of Public Health, College of Health Sciences<br>Kiwanuka, Suzanne ; Makerere University, Department of Health Policy, Planning and Management, School of Public Health, College of Health Sciences<br>Sambisa, William; Bill & Melinda Gates Foundation<br>Tsai, Lily ; Massachusetts Institute of Technology<br>Wanyenze, Rhoda K; Makerere University, 1. Department of Disease Control and Environmental Health, School of Public Health, College of Health Sciences
**Primary Subject Heading**:	Public health
Secondary Subject Heading:	Infectious diseases, Public health
Keywords:	COVID-19, PUBLIC HEALTH, INFECTIOUS DISEASES

SCHOLARONE™
Manuscripts

I, the Submitting Author has the right to grant and does grant on behalf of all authors of the Work (as defined in the below author licence), an exclusive licence and/or a non-exclusive licence for contributions from authors who are: i) UK Crown employees; ii) where BMJ has agreed a CC-BY licence shall apply, and/or iii) in accordance with the terms applicable for US Federal Government officers or employees acting as part of their official duties; on a worldwide, perpetual, irrevocable, royalty-free basis to BMJ Publishing Group Ltd ("BMJ") its licensees and where the relevant Journal is co-owned by BMJ to the co-owners of the Journal, to publish the Work in this journal and any other BMJ products and to exploit all rights, as set out in our licence.

The Submitting Author accepts and understands that any supply made under these terms is made by BMJ to the Submitting Author unless you are acting as an employee on behalf of your employer or a postgraduate student of an affiliated institution which is paying any applicable article publishing charge ("APC") for Open Access articles. Where the Submitting Author wishes to make the Work available on an Open Access basis (and intends to pay the relevant APC), the terms of reuse of such Open Access shall be governed by a Creative Commons licence – details of these licences and which Creative Commons licence will apply to this Work are set out in our licence referred to above.

Other than as permitted in any relevant BMJ Author's Self Archiving Policies, I confirm this Work has not been accepted for publication elsewhere, is not being considered for publication elsewhere and does not duplicate material already published. I confirm all authors consent to publication of this Work and authorise the granting of this licence.

For peer review only

Uptake of COVID-19 vaccines and associated factors among adults in Uganda: a cross-sectional survey

Rawlance Ndejjo[1]*, Nuole Chen[2], Steven N. Kabwama[3], Alice Namale[1], Solomon T. Wafula[1], Irene Wanyana[4], Susan Kizito[1], Suzanne Kiwanuka[5], William Sambisa[6], Lily L. Tsai[2], Rhoda K. Wanyenze[1]

Affiliations

1. Department of Disease Control and Environmental Health, School of Public Health, College of Health Sciences, Makerere University, Kampala, Uganda
2. Massachusetts Institute of Technology, Massachusetts Avenue, Cambridge, USA
3. Department of Community Health and Behavioral Sciences, Makerere University School of Public Health, Kampala, Uganda
4. Department of Epidemiology and Biostatistics, School of Public Health, College of Health Sciences, Makerere University, Kampala, Uganda
5. Department of Health Policy, Planning and Management, School of Public Health, College of Health Sciences, Makerere University, Kampala, Uganda
6. Bill & Melinda Gates Foundation, Seattle, Washington, USA

* Correspondence, Rawlance Ndejjo

Department of Disease Control and Environmental Health, School of Public Health, College of Health Sciences, Makerere University, Kampala, Uganda, P.O Box 7072, Kampala, Uganda

Email: rndejjo@musph.ac.ug

For peer review only - http://bmjopen.bmj.com/site/about/guidelines.xhtml

Abstract

Objective The Coronavirus Disease (COVID-19) pandemic remains one of the most significant public health challenges ever faced globally. Vaccines are key to ending the pandemic as well as minimize its consequences. This study determined the uptake of COVID-19 vaccines and associated factors among adults in Uganda.

Design, setting and participants We conducted a cross-sectional mobile phone survey among adults in Uganda.

Main outcome variable Participants reported their uptake of COVID-19 vaccines.

Results Of the participants contacted, 94% (1173) completed the survey. Overall, 49.7% had received COVID-19 vaccines with 19.2% having obtained a full dose and 30.5% an incomplete dose. Among the unvaccinated, 91.0% indicated intention to vaccinate. Major reasons for vaccine uptake were protection of self from COVID-19 (86.8%) and a high perceived risk of getting the virus (19.6%). On the other hand, non-uptake was related to vaccine unavailability (42.4%), lack of time (24.1%), and perceived safety (12.5%) and effectiveness concerns (6.9%). The factors associated with receiving COVID-19 vaccines were older age (≥ 65 years) (APR = 1.32 (95% CI: 1.08 – 1.61), secondary (APR = 1.36 (95% CI: 1.12 – 1.65), or tertiary education (APR = 1.62 (95% CI: 1.31 – 2.00), and health workers as a source of information on COVID-19 (APR = 1.26 (95% CI: 1.10 – 1.45). Also, reporting a medium-income (APR = 1.24 (1.02 – 1.52) and residence in Northern (APR = 1.55, 95% CI 1.18 – 2.02) and Central regions (APR = 1.48, 95% CI 1.16 – 1.89) were associated with vaccine uptake.

Conclusions Uptake of COVID-19 vaccines was moderate in this sample and was associated with older age, secondary and tertiary education, medium-income, region of residence, and

For peer review only

For peer review only - http://bmjopen.bmj.com/site/about/guidelines.xhtml

health workers as a source of COVID-19 information. Efforts are needed to increase access to vaccines and should utilize health workers as champions to enhance uptake.

Wordcount: 3300

Keywords: associated factors, COVID-19, intention, uptake, Uganda, vaccines, willingness

STRENGTHS AND LIMITATIONS OF THIS STUDY

- This study had a high response rate with over 94% of the participants consenting to participate in the phone survey.
- Results from the backchecking with the same individuals showed high consistency with the survey results.
- Being a mobile phone survey, the study participants were not representative of the population as only those with a mobile phone could participate.
- Reporting of vaccination status could have been subject to social desirability bias, which we minimized by reminding participants that the study was only for research purposes.

For peer review only - http://bmjopen.bmj.com/site/about/guidelines.xhtml

INTRODUCTION

The Coronavirus disease of 2019 (COVID-19) has resulted in significant morbidity and mortality globally and negatively disrupted multiple socio-economic sectors. As of 31st March 2022, over 488 million confirmed cases and 6.1 million deaths had been registered globally [1]. In Africa, more than 11 million confirmed COVID-19 cases and 251,953 deaths were reported since the onset of the epidemic. Within the same period, Uganda recorded 163,905 cumulative COVID-19 cases and 3,596 confirmed deaths [1]. In response and under the advice of the World Health Organization, many countries at the beginning of the pandemic implemented non-pharmaceutical interventions (NPIs) that restricted movement such as lockdowns and curfews. Several governments both globally and in Africa also closed schools, places of worship, recreation centres, and public places. Governments also promoted regular hand and respiratory hygiene, wearing of facemasks, ensuring physical and social distancing, and working from home [2]. These public health and social measures significantly impacted the delivery of routine health care services, caused job losses, disrupted education and formal and informal trade, and increased gender-based violence and mental health disorders [3-5].

Vaccines as key pharmaceutical interventions to contain COVID-19 were adopted almost one year into the pandemic globally. Uganda recorded its first confirmed case of COVID-19 on 21st March 2020 and received its first batch of COVID-19 vaccines one-year later in March 2021. At the start, vaccination targeted high-risk groups including health workers, teachers, security personnel, persons older than 50 years, and those with co-morbidities. Starting August 2021 when the country received more doses of vaccines, vaccination was opened up to all Ugandans aged 18 years and above. Vaccines were largely available through designated health facilities, outreaches and mobile vaccination service points. The Ministry of Health (MoH) ran media

For peer review only

For peer review only - http://bmjopen.bmj.com/site/about/guidelines.xhtml

campaigns to mobilise communities for COVID-19 vaccination working hand in hand with local government structures. High vaccination coverage was critical for containment of the pandemic, re-opening of the economy and reversal of the negative socio-economic impacts of the NPIs. However, the opening up of eligibility for vaccination was marred with negative information and fears of vaccine hesitancy. In order to develop critical strategies to achieve high vaccination coverage, there is need for an in-depth understanding of factors influencing the uptake of COVID-19 vaccination. This study, therefore, sought to gather and analyse data to determine the uptake of COVID-19 vaccines and associated factors among adults in Uganda.

METHODS

Study setting

This study was conducted in Uganda located in Eastern Africa. The country has 136 districts distributed in four administrative regions (Northern, Eastern, Central, and Western) which were all involved in the study. As of 2020, Uganda had an estimated population of approximately 41.8 million people [6]. Having registered its first confirmed case of COVID-19 in March 2020, the country had by November 2021 experienced two waves of the disease. The first wave of the pandemic occurred from August 2020 to February 2021 of various non-Delta variants while the second wave happened from May to October 2021 fueled by the Delta variant [1, 7].

Study design and population

This was a cross-sectional mobile phone survey conducted in November 2021 among a nationally constituted sample of adults. The study enrolled persons aged ≥18 years sampled from the country's four administrative regions: Central, Eastern, Northern and Western. We excluded persons who said they were ill and unable to participate in the interview.

For peer review only - http://bmjopen.bmj.com/site/about/guidelines.xhtml

Sample size estimation

To enable tracking changes in adherence to NPIs following the introduction of vaccines, we used a previous sample of study respondents from an earlier survey [8] whose data were collected in March 2021 [8]. The sample size for the previous survey was determined using the Leslie Kish formula for cross-sectional studies [9] considering the following assumptions: Two-sided Z statistic corresponding to a 95% confidence interval (1.96), NPI adherence level of 50% since no other study had been conducted to show the composite level of adherence, a precision of 5% and a design effect of 2.5 [8]. Considering a non-response rate of 10%, the total sample size estimate was 1056 people.

Sampling strategy

We used the sample from an earlier survey [8], which was constituted following quota sampling. Quotas were set on age, sex, and location proportionate to national COVID-19 case distribution statistics as of February 2021 [10]. The distribution of cases at the time was as follows: age: 18–35 years (51%), 36–55 years (37%), 56–65 years (8%), 65+ years (4%); sex: male (60%) and female (40%); and region: Central (55%) and 15% for each of Eastern, Western, and Northern regions. With quotas in place, a simple randomly selected (SRS) sample was obtained among the eligible population using a database of phone contacts provided by a registered research firm. In cases of replacement of previous participants due to unavailability or refusal to participate, a similar case distribution was followed during sampling of new contacts.

Data collection

Data were collected using a structured survey questionnaire (supplementary file 01), with mostly closed-ended questions, informed by a review of published literature [8, 11, 12]. The

For peer review only - http://bmjopen.bmj.com/site/about/guidelines.xhtml

questionnaire was pretested among 20 people from the four regions of Uganda and relevant adjustments were made. The questionnaire was translated into nine major local languages spoken in Uganda, namely: *Ateso, Luganda, Lugbara, Lugisu, Luo, Lusoga, Ngakarimojong, Runyankole-Rukiga* and *Runyoro-Rutooro.* An independent group of translators validated the questionnaire translations and any discrepancies were addressed. The final survey instrument in each language was programmed in SurveyCTO software, incorporating appropriate routing, conditional logic, and other controls and uploaded on hand-held mobile tablets. Bench testing of the survey questionnaire was conducted, and adjustments made before actual data collection. Trained research assistants with a minimum of a Diploma in a health-related field, fluent in the survey languages and with experience in mobile surveys conducted the interviews. Research assistants made phone calls from a designated place in Kampala to the respondents from whom they sought verbal informed consent after explaining to them what the study entailed and entered data into the tablets. The average interview time was 26 minutes. Respondents who preferred to defer the phone interviews due to busy schedules or other reasons received follow-up phone calls based on agreed-upon appointment times. Daily checks of the survey data were conducted to monitor quality and intervene early and appropriately, as well as ensure adherence to established quotas. A team of supervisors oversaw the work of the research assistants ensuring that questions were asked appropriately, and respondents were interviewed in the language they were most comfortable with. At the end of the interview period, we conducted back checking of 10% of respondents to ascertain the quality of collected data.

Data management and analysis strategy

During data collection, each research assistant examined, edited, and cleaned their data daily before uploading it to the server. Data were encrypted and anonymized on the server and later

For peer review only - http://bmjopen.bmj.com/site/about/guidelines.xhtml

downloaded and exported to Stata 15.0 for further cleaning. Data analysis was conducted in Rstudio Version 1.4.1106 (RStudio, PBC). Descriptive statistics have been provided in the form of means (standard deviation) for continuous variables while categorical variables have been expressed as frequencies and percentages. Socioeconomic status was generated as an additive index from 6 variables on household ownership of television, computer, sofa set, refrigerator, and cassette/CD/DVD player, and access to electricity. The socio-economic status index was then divided into tertiles. The dependent variable was self-reported uptake of COVID-19 vaccines, which constituted those who reported receiving at least one dose of any World Health Organization (WHO) approved COVID-19 vaccines. We also determined the intention to uptake COVID-19 vaccines by asking unvaccinated respondents if they intended to receive the vaccine. The independent variables included socio-demographic characteristics (age, gender, employment status, education and occupation, place of residence (urban vs. rural, region) and source of information on COVID-19. To determine the factors associated with vaccination uptake, we ran multivariable modified Poisson regressions with robust error variance and presented prevalence ratios and corresponding 95% confidence intervals. Only variables with a p-value ≤ 0.2 at bivariate levels were included in the final model.

Patient and public involvement

No patients or members of the public were involved in the study design, setting the research questions, interpretation or writing up of results, or reporting of the research.

For peer review only - http://bmjopen.bmj.com/site/about/guidelines.xhtml

RESULTS

Sociodemographic characteristics of participants

Of the 1,249 respondents reached, a total of 1,173 (94%) participants completed the survey. The mean age of respondents was 39.7 years (SD ±14.2) and majority 717 (61.1%) were males. Half 606 (51.7%) of the study participants were from the Central region, 548 (46.8%) had an urban residence and 548 (46.7%) belonged to the lowest socioeconomic tertile. Nearly four in ten (39%) respondents had only primary or no formal education (Table 1).

Table 1: Sociodemographic characteristics of participants

Characteristic	Number of participants (N = 1,173)	Percentage (%)[1]
**Sex**		
Male	717	61.1
Female	456	38.9
**Age group (years), Mean age (SD)**	39.7 (±14.2)	
18 – 35	553	47.1
36 – 55	439	37.4
56 - 64	92	7.8
65+	89	7.6
**Region of residence**		
North	182	15.5
East	211	18
Central	606	51.7
West	174	14.8
**Residence**		
Urban	548	46.8
Rural	417	35.6
Semi-urban	207	17.7
Not stated	1	
**Earnings per month ($)**		
< 14	256	25.6
14 – 29	226	22.6
30 – 57	196	19.6
58 – 143	229	22.9
> 143	93	9.3

For peer review only

For peer review only - http://bmjopen.bmj.com/site/about/guidelines.xhtml

Characteristic	Number of participants (N = 1,173)	Percentage (%)[1]
Not stated	173	
**Education level**		
None or Incomplete Primary	265	23.2
Primary (completed)	180	15.7
Secondary	431	37.7
Tertiary	268	23.4
Not stated	27	
**Socio-economic index**		
Low	548	46.7
Middle	435	37.1
Higher	190	16.2
**Religion**		
Catholic	384	33.3
Anglican	372	32.3
Born Again (Pentecostal)	147	12.8
Muslim	226	19.6
Other religions	24	2.1
Not stated	20	
**Current Occupation**		
Unemployed	193	17.1
Employed	182	16.1
Self employed	355	31.4
Casual laborer	67	5.9
Farmer	334	29.5
Not stated	42	
**Current household size, Mean (SD)**	5.6 (3.5)	
5 or fewer	653	55.7
6 – 10	430	36.7
More than 10	90	7.7

[1] *Percentages calculated do not include respondents who did not record responses (e.g. "Not stated" in the tables)*

For peer review only

Uptake of COVID-19 vaccines and intention to vaccinate

Among all respondents, 225 (19.2%) reported receiving a full dose of the vaccine and 357 (30.5%) an incomplete dose. Slightly above sixty per cent of the respondents 367 (63.2%) reportedly experienced side effects following vaccination mostly fever 147 (40.1%), fatigue 115

(31.3%) and headache 101 (27.5%). Among those who had not received a vaccine, 537 (91.8%) reported intention to vaccinate (

Table 2).

Table 2: Vaccination uptake and intention to vaccinate among participants

Variable	Count	Percentage (%)[1]
**Vaccination uptake**	**(n = 582)**	
Full dose (two shots)	225	19.2
Incomplete dose	357	30.5
No vaccination	590	50.3
**Experienced any side effects after first dose**		
No	214	36.8
Yes	367	63.2
**Side effects reported**	**(n = 367)**	
Fever	147	40.1
Fatigue	115	31.3
Headache	101	27.5
Muscle soreness /pain	95	25.9
Injection site reaction	88	24.0
Others[2]	38	10.4
**Vaccination intention (among unvaccinated)**	**n = 590**	
Intend to vaccinate	537	91.0
Did not intend to vaccinate	48	8.1
Did not know	5	0.8

[1] Percentages calculated do not include respondents who did not record responses e.g. "Not stated" in the tables
[2] Allergic reaction, cough, body pain, dizziness, arrhythmias, body weakness, paralysis for a few days, erectile dysfunction for a few days

For peer review only - http://bmjopen.bmj.com/site/about/guidelines.xhtml

For peer review only

Reasons for vaccine uptake/ non-uptake and intention/un intention to vaccinate

The reasons for COVID-19 vaccine uptake and intention to vaccinate were similar with both categories of respondents mostly reporting the need to obtain protection from COVID-19 and having a high perceived risk of getting the virus. Over 40% of respondents who had not been vaccinated attributed it to vaccine unavailability 250 (42.4%) and below a quarter of respondents to not having time 142 (24.1%). The reasons for lack of intention to vaccinate were mainly related to safety 24 (50.0%) and effectiveness concerns 17 (35.4%) which were similarly reported for non-uptake of vaccines (

For peer review only

Table 3).

Table 3: Reasons for (non) uptake of COVID-19 vaccines and intention to vaccinate (multiple response)

Reasons	Uptake of vaccines n = 582 (%)	Intention to vaccinate n = 537 (%)
To protect self from COVID-19	505 (86.8%)	458 (85.3%)
High perceived risk of getting COVID-19	114 (19.6)	90 (16.8%)
Prioritized due to health (comorbidities)	95 (16.3)	34 (6.3%)
Recommendation from health workers	81 (13.9)	38 (7.1)
Prioritized due to occupation	74 (12.7)	-
Travel purposes	44 (7.6)	45 (8.4%)

For peer review only - http://bmjopen.bmj.com/site/about/guidelines.xhtml

Job requirement	-	82 (15.3%)
Others	21 (3.6)[1]	20 (3.8)[2]
**Reasons**	**Non uptake of vaccines n = 590 (%)**	**No intention to vaccinate n = 48 (%)**
Vaccines are unavailable	250 (42.4)	1 (2.1)
Don't have time	142 (24.1)	2 (4.2)
Safety concerns	74 (12.5)	24 (50.0)
Doubt vaccine effectiveness	41 (6.9)	17 (35.4)
Not among eligible group	30 (5.1)	4 (8.3)
Transport costs	24 (4.1)	Not reported
Don't know where to access the vaccines from	20 (3.4)	Not reported
Do not fear COVID-19/ trust immunity	10 (1.7)	1 (2.1)
Others	82 (14.0)[3]	7 (14.6)[4]

[1] Requirement for school attendance, being exemplary, following MOH guidelines, boosting immunity, to access services, among the eligible group.
[2] Access to health services, government mandate, pressure from peers, to be exemplary, requirement for school attendance.
[3] Pregnant, breastfeeding, waiting for another vaccine type, lack identification documents, long queues, currently sick, recently recovered from COVID-19.
[4] Religious beliefs, do not believe COVID-19, HIV positive and fear side effects, underlying Hepatitis B infection, Body already weak, Lack of identification documents.

Willingness to vaccinate for different vaccine types

All respondents were asked if they would receive the different types of COVID-19 vaccines if offered at that point and were free of charge. Only 316 (26.9%) reported that they would take any vaccine regardless of the type and 488 (41.6%) indicated a willingness to take at least one type of the vaccine. The most preferred COVID-19 vaccine types were Johnson and Johnson 436 (37.4%) and AstraZeneca 405 (34.7%) (Figure 1).

Figure 1 Willingness for COVID-19 vaccination for different vaccine types

For peer review only - http://bmjopen.bmj.com/site/about/guidelines.xhtml

Factors associated with uptake of COVID-19 vaccines

At the multivariable analysis level, participants aged ≥65 years had a 32% higher likelihood to have been vaccinated compared to those aged 18-35 years (Adjusted PR = 1.32, 95% CI 1.08 – 1.61, p = 0.008). Participants from the Northern (adjusted PR = 1.55, 95% CI 1.18 – 2.02, p = 0.002) and Central regions (adjusted PR = 1.48, 95% CI 1.16 – 1.89, p = 0.002) respectively had a 55% and 48% higher likelihood to have received the vaccine compared to those from the Western region. Participants with secondary (adjusted PR = 1.36, 95% CI 1.12 – 1.65, p = 0.002) or tertiary education (Adjusted PR = 1.62, 95% CI 1.31 – 2.00, p < 0.001) were more likely to have received the COVID-19 vaccine compared to those with incomplete primary/no formal education. Respondents whose monthly income was between $30 and $57 (APR = 1.24 (1.02 – 1.52), p = 0.029) had a higher uptake of COVID-19 vaccines than those who earned < $14. Having health workers as a source of information on COVID-19 was associated with higher uptake of COVID-19 vaccines in Uganda (adjusted PR = 1.26, 95%CI 1.10 – 1.45, p = 0.001) (Table 4).

Table 4: Factors associated with COVID-19 vaccine uptake among adults

Variables / Characteristics	Self-reported uptake of COVID-19 vaccine		Unadjusted PR (95% CI)[1]	p-value	Adjusted PR (95% CI)[2]	p-value
	No (%)	Yes (%)				
**Age in years**						
18 – 35	296 (53.5)	257 (46.5)	1		1	
36 – 55	213 (48.6)	225 (51.4)	1.11 (0.97 – 1.26)	0.124	1.09 (0.95 – 1.25)	0.244
56 – 64	44 (47.8)	48 (52.2)	1.12 (0.91 – 1.39)	0.292	1.17 (0.92 – 1.48)	0.193
65+	37 (41.6)	52 (58.4)	1.26 (1.03 -1.53)	**0.023**	1.32 (1.08 – 1.61)	**0.008**

For peer review only - http://bmjopen.bmj.com/site/about/guidelines.xhtml

Variables / Characteristics	Self-reported uptake of COVID-19 vaccine		Unadjusted PR (95% CI)[1]	p-value	Adjusted PR (95% CI)[2]	p-value
	No (%)	Yes (%)				
**Region of residence**						
Western	115 (66.5)	58 (33.5)	1		1	
Northern	74 (40.7)	108 (59.3)	1.77 (1.39 – 2.25)	**<0.001**	1.55 (1.18 - 2.02)	**0.002**
Eastern	112 (53.1)	99 (46.9)	1.40 (1.09 – 1.80)	**0.010**	1.29 (0.99 - 1.69)	0.064
Central	289 (47.7)	317 (52.3)	1.56 (1.25– 1.95)	**0.001**	1.48 (1.16 - 1.89)	**0.002**
**Residence**						
Urban	270 (49.4)	277 (50.6)			1	
Rural	206 (49.4)	211 (50.6)	0.99 (0.88 – 1.13)	0.990	1.11 (0.97 – 1.28)	0.137
Semi-urban	114 (55.1)	93 (44.9)	0.89 (0.75 – 1.05)	0.173	0.92 (0.75 – 1.11)	0.373
**Gender**						
Male	351 (49.0)	366 (51.0)	1		1	
Female	239 (52.5)	216 (47.5)	0.93 (0,82 – 1.05)	0.237	1.00 (0.87 – 1.14)	0.973
**Wealth index**						
Low	290 (53.0)	257 (47.0)	1		1	
Middle	217 (49.9)	218 (50.1)	1.07 (0.94 – 1.21)	0.328	1.06 (0.91 – 1.24)	0.442
High	83 (43.7)	107 (56.3)	1.20 (1.03 – 1.40)	**0.021**	1.03 (0.83 – 1.28)	0.758
**Current Occupation**						
Unemployed	91 (47.2)	102 (52.8)	1		1	
Employed	76 (41.8)	106 (58.2)	1.10 (0.92 – 1.32)	0.294	1.03 (0.84 – 1.27)	0.763
Self employed	196 (55.2)	159 (44.8)	0.85 (0.71 – 1.01)	0.066	0.84 (0.68 – 1.02)	0.078
Casual laborer	45 (67.2)	22 (32.8)	0.62 (0.43 – 0.90)	**0.011**	0.73 (0.48 – 1.11)	0.146
Farmer	164 (49.1)	170 (50.9)	0.96 (0.81 – 1.14)	0.664	0.99 (0.82 – 1.19)	0.931
**Education level**						
No formal education /	161 (60.8)	104 (39.2)	1		1	

For peer review only - http://bmjopen.bmj.com/site/about/guidelines.xhtml

Variables / Characteristics	Self-reported uptake of COVID-19 vaccine		Unadjusted PR (95% CI)[1]	p-value	Adjusted PR (95% CI)[2]	p-value
	No (%)	Yes (%)				
incomplete primary						
Complete primary	109 (60.6)	71 (39.4)	1.01 (9.79 - 1.27)	0.966	1.00 (0.78 – 1.28)	0.998
Secondary education	207 (48.0)	224 (52.0)	1.32 (1.11 – 1.58)	**0.002**	1.36 (1.12 – 1.65)	**0.002**
Tertiary	98 (36.6)	170 (63.4)	1.62 (1.36 – 1.93)	**< 0.001**	1.62 (1.31 – 2.00)	**< 0.001**
**Household size (mean)**	5.41	5.99	1.02 (1.01 – 1.03)	**< 0.001**	1.02 (1.00 – 1.03)	0.071
**Monthly income ($)**						
< 14	144 (56.2)	112 (43.8)	1		1	
14 – 29	117 (51.8)	109 (48.2)	1.10 (0.91 – 1.34)	0.324	1.08 (0.89 – 1.32)	0.423
30 – 57	86 (43.9)	110 (56.1)	1.28 (1.07 – 1.55)	**0.009**	1.24 (1.02 – 1.52)	**0.029**
58 – 143	114 (49.8)	115 (50.2)	1.15 (0.95 – 1.39)	0.154	0.98 (0.79 – 1.22)	0.876
> 143	36 (38.7)	57 (61.3)	1.40 (1.13 – 1.73)	**0.002**	1.16 (0.91 – 1.49)	0.219
**Health workers as source of information on COVID-19[3]**						
No	245 (57.9)	178 (42.1)	1		1	
Yes	345 (46.1)	404 (53.9)	1.28 (1.13 – 1.45)	**< 0.001**	1.26 (1.10 – 1.45)	**0.001**

[1] Bivariate analysis

[2] Multivariable analysis

[3]Other sources of information included family members, friends/peers, Radio, Television, community members and social media among others which were dichotomized and included in the analysis but were not significant

DISCUSSION

This study examined the uptake of COVID-19 vaccines and associated factors among adults aged 18 years and above in Uganda. Among the study participants, about one in five (19.2%) reported receiving a full dose of the COVID-19 vaccine while 30.5% had received an incomplete dose. Over 90% of those who were unvaccinated reported the intention to be vaccinated. The

For peer review only

major reasons for vaccine uptake and intention to vaccinate were protection of self from COVID-19 and a high perceived risk of getting the virus while reasons for vaccine non-uptake were vaccine unavailability, the lack of time to go get vaccinated, and safety and effectiveness concerns. The factors that were associated with receiving the COVID-19 vaccine were older age (65 years and above), having secondary education and above, having a moderate income, and reporting health workers as a source of information on COVID-19. Being a resident of Northern and Central Uganda was also associated with a high likelihood of receiving the vaccine.

Uptake of COVID-19 vaccines in this sample of respondents was higher for both full and incomplete doses than the vaccinated proportion of the population as of November 2021 when this study was conducted. Ministry of Health data of 8th November 2021 indicated that 55.8% and 16.8% of the priority groups and 12.2% and 3.7% of the adult population had received their first and second doses of the vaccine respectively [13]. The higher-than-baseline vaccination coverage could be attributed to the use of mobile phones for the survey and thus the relatively urbanized study sample whose access to vaccines was higher than those in rural areas. Moreover, a high proportion of participants were from the Central region, which was most impacted by COVID-19, and their experiences could have influenced vaccine uptake. In addition, intention to vaccinate was very high at over 90%; higher than the combined “definite intention” of 57.8% and “probable intention” of 26.2% from the March survey round [8]. In a November 2021 survey among 23,000 respondents from 19 African Union members states including Uganda, (78%) of respondents had either been vaccinated or were likely to get vaccinated [14]. The second COVID-19 wave fueled by the Delta variant that was experienced in Uganda from June to September 2021 and led to at least 2,800 deaths compared to the less than 300 recorded at the end of the first wave [1, 15] could also have contributed to the high uptake of the vaccine and

For peer review only - http://bmjopen.bmj.com/site/about/guidelines.xhtml

intention-to-vaccinate. In addition, there was concern about potential vaccine mandates including anticipation that the unvaccinated would be denied health and social services which could also have increased the intention to vaccinate.

The major reasons for vaccine uptake and intention to vaccinate were protection of self from COVID-19 and a high perceived risk of getting the virus, similar to previous research [11]. This is also an indication of the respondents' appreciation of the role of vaccines in preventing morbidity and saving lives. Those unvaccinated attributed it to vaccine unavailability and the lack of time. The survey in 19 African countries concluded that low vaccine uptake was mostly due to unpredictable supply of vaccines and logistical hurdles than reluctance or refusal to get vaccinated [14]. Earlier surveys in Uganda conducted in 2020 had also shown a high acceptance of COVID-19 vaccines of over 85% [11, 16]. To bridge the willingness-intention-uptake gap in Uganda, the Ministry of Health should increase access and availability of COVID-19 vaccines. Evidence shows that strategies that take vaccines closer to the communities are likely to mitigate time and transport-related barriers and increase vaccine uptake [17, 18]. This could be achieved by increasing the number of health facilities offering the vaccines, conducting more vaccination outreaches, or setting up mobile vaccine points. The World Health Organization guidance has also emphasized the importance of location and time in COVID-19 vaccine uptake [19]. On the other hand, the study reported that safety and effectiveness concerns hindered vaccine uptake and intention to vaccinate similar to previous research [8, 11, 20-23]. Of note as well was the observed high prevalence (63%) of self-reported vaccine side effects which could go a long way in reinforcing safety concerns among the population. Vaccine adverse events should be monitored closely, and appropriate information, education and communication material developed including information on expected side effects to counter their potential effect on the

For peer review only

uptake of vaccination by the unvaccinated. Accurate, consistent and transparent communication and dialogue about uncertainty, risks and anticipated benefits can go a long way in building confidence and trust in the COVID-19 vaccines and create motivation for vaccination [19, 23]. This could also bridge observed gaps in vaccine preference to prevent this from being a barrier to vaccination. The Johnson and Johnson vaccine being a single shot had a higher preference among respondents due to the perceived inconvenience and unpredictability of obtaining a second vaccine dose.

It was not surprising that those aged 65 years and above had a higher vaccination uptake as these were part of the prioritized group for COVID-19 vaccination in the country. Education status also predicted vaccination status similar to previous research on COVID-19 vaccine acceptability [24-27]. However, further efforts are required to ensure the dissemination of accurate and simple COVID-19 vaccination messages to those of lower education levels including translating information in the local languages so that this group is not left behind. A moderate income was associated with higher vaccine uptake; however, this relationship was not sustained with increasing income levels. The regional differences observed in the uptake of COVID-19 vaccines may have been due to differences in vaccine access and availability, especially for Central region which was most hit by the pandemic and was prioritized early during vaccine rollout. From previous research, income levels and locations have been reported as predictors of COVID-19 vaccine acceptability [27, 28].

One major finding from our work was that respondents whose source of information on COVID-19 was health workers had a higher likelihood for COVID-19 vaccination. This positions health workers as a key resource in increasing vaccination uptake, and thus they should be furnished with sufficient and accurate information and supported with effective communication tools to

For peer review only - http://bmjopen.bmj.com/site/about/guidelines.xhtml

influence their clients at facility and community level. Previous studies report that health worker advice on vaccination was most trusted [11, 22]. Health workers can lead health education and awareness programs on COVID-19 and use their platforms at health facility and community level to influence the masses to uptake COVID-19 vaccines. However, vaccine uptake among health workers themselves was low at the time even when they were prioritized for vaccination from the start of the campaigns in Uganda and elsewhere. In a March 2021 survey in Uganda, just after the launch of the COVID-19 vaccination exercise, a vaccine acceptability rate of 37.3% and hesitancy of 30.7% were reported among medical students [12]. In a June to August 2021 online survey, acceptance or willingness to uptake the COVID-19 vaccine stood at over 97% and 65.3% of eye healthcare workers had received a COVID-19 vaccine shot influenced by high perceived susceptibility and benefits [29]. An in-depth study among health workers reported the lack of trust in the vaccine, fear of side effects, not feeling at risk, lack of sufficient information about vaccines, health systems challenges and religious beliefs as barriers to COVID-19 vaccination [30]. When health workers are vaccinated, they are more likely to recommend the same to their clients [31]. Therefore, appropriate interventions should be instituted to effectively deal with vaccine hesitancy among health workers and have them as champions for COVID-19 vaccination.

Study limitations and strengths

Being a mobile phone survey, the study participants were not representative of the population and only those with a mobile phone could participate, contributing to selection bias. However

For peer review only

For peer review only - http://bmjopen.bmj.com/site/about/guidelines.xhtml

mobile phone coverage in Uganda has increased over the years; according to the Uganda National Household Survey 2020, 74.0% of Ugandans own mobile phones [32]. There was also potential for social desirability bias, especially regarding reporting vaccination status which we minimized by reminding participants that the study was only for research purposes. Also, as a cross-sectional survey, the direction of associations observed is not clear. On the other hand, our study had a high response rate with over 94% of the participants consenting to participate. The high response rate could be attributed to following up previous survey participants, flexibility in conducting interviews at convenient times, as well as the time compensation (phone credit of 1.5 US dollars) provided. Results from the backchecking with the same individuals also showed high consistency with the survey results. Our study provides insights into COVID-19 vaccination uptake and intention to vaccinate which can facilitate the development of context-relevant strategies to increase vaccinations.

CONCLUSIONS

Half of the study respondents were vaccinated against COVID-19, which was associated with older age, higher education level, moderate income, region of residence and reporting health workers as the source of COVID-19 information. Among the unvaccinated, over 90% expressed intention to vaccinate. Efforts are needed to increase access to vaccines and utilize health workers as a key resource in sharing information and champions to influence the masses which should positively impact uptake of COVID-19 vaccines.

Acknowledgements

For peer review only - http://bmjopen.bmj.com/site/about/guidelines.xhtml

The authors wish to thank the study participants who provided the information that led to this publication. The research assistants are also appreciated for their contribution to this study.

Author Contributions

RN, NC, SNK, AN, WS, LLT and RKW conceptualized and designed the study. RN, NC, SNK, AN, IW, SKiz supported the data collection. RN, NC, SNK, AN, STW, IW, SKiz, SKiw, WS, LLT and RKW contributed to analysis and interpretation of findings. RN, NC, STW wrote the first draft of the manuscript. SNK, AN, IW, SKiz, SKiw, WS, LLT and RKW critically reviewed the draft manuscript. All authors read and approved the final manuscript.

Funding

This work was supported in whole or in part, by the Bill & Melinda Gates Foundation [Opportunity ID: INV-019313]. The views, opinions, and content of this publication are those of the authors and do not necessarily reflect the views, opinions, or policies of the Bill and Melinda Gates Foundation.

Competing interests

None declared.

Consent for publication

Not applicable

For peer review only - http://bmjopen.bmj.com/site/about/guidelines.xhtml

Ethics approval and consent to participate

Ethical approval to conduct the survey was sought from the Makerere University School of Public Health Higher Degrees Research and Ethics Committee (protocol SPH-2021-150) and the study was registered by the Uganda National Council of Science and Technology (HS1742ES). Verbal consent was provided before participation in the survey. Personally identifiable information including name of respondent, their phone number and the household head name were encrypted with passwords on the SurveyCTO server and drives and was only accessible to the investigator(s). All phones and tablets used for data collection were password-protected to protect respondent data.

Data availability statement

The data are available from the corresponding author on reasonable request.

Supplementary files

Supplementary file 01: Study questionnaire

REFERENCES

1. WHO. COVID-19 Coronavirus Pandemic: World Health Organization; 2021 [Available from: https://www.worldometers.info/coronavirus/#countries accessed 22nd January 2021.
2. WHO. Modes of transmission of virus causing COVID-19: implications for IPC precaution recommendations: scientific brief, 27 March 2020: World Health Organization, 2020.

For peer review only - http://bmjopen.bmj.com/site/about/guidelines.xhtml

3. WHO. COVID-19 significantly impacts health services for noncommunicable diseases. 2020 2020 [Available from: https://www.who.int/news-room/detail/01-06-2020-covid-19-significantly-impacts-health-services-for-noncommunicable-diseases.
4. Vindegaard N, Benros ME. COVID-19 pandemic and mental health consequences: Systematic review of the current evidence. *Brain, behavior, and immunity* 2020;89:531-42.
5. Piquero AR, Jennings WG, Jemison E, et al. Domestic violence during the COVID-19 pandemic - Evidence from a systematic review and meta-analysis. *Journal of Criminal Justice* 2021;74:101806. doi: https://doi.org/10.1016/j.jcrimjus.2021.101806
6. Uganda Bureau of Statistics. National Population Projections by single age and sex (2015–2040). Kampala: Uganda Bureau of statistics; 2020 [accessed 12th August 2021.
7. Elayeete S, Nampeera R, Nsubuga EJ, et al. Comparative epidemiologic analysis of COVID-19 patients during the first and second waves of COVID-19 in Uganda. *IJID Regions* 2022;3:160-67. doi: https://doi.org/10.1016/j.ijregi.2022.03.017
8. Wafula ST, Mugume IB, Sensasi B, et al. Intention to vaccinate against COVID-19 and adherence to non-pharmaceutical interventions against COVID-19 prior to the second wave of the pandemic in Uganda: a cross-sectional study. *BMJ Open* 2022;12(6):e057322. doi: 10.1136/bmjopen-2021-057322 [published Online First: 2022/06/03]
9. Kish L. Survey sampling. Oxford, England: Wiley 1965.
10. Uganda Ministry of Health. National COVID-19 surveillance data, February 2021. : Ministry of Health, 2021.
11. Solís Arce JS, Warren SS, Meriggi NF, et al. COVID-19 vaccine acceptance and hesitancy in low-and middle-income countries. *Nature medicine* 2021;27(8):1385-94.
12. Kanyike AM, Olum R, Kajjimu J, et al. Acceptance of the coronavirus disease-2019 vaccine among medical students in Uganda. *Tropical Medicine and Health* 2021;49(1):37. doi: 10.1186/s41182-021-00331-1
13. Uganda Ministry of Health. COVID-19 Vaccination Update 10th November 2021. Kampala, Uganda: Ministry of Health, 2021.
14. (PERC) PfE-BRtC-. Responding to COVID-19 in Africa Finding the Balance Part IV, 2021.
15. World Health Organization. Uganda: WHO Coronavirus Disease (COVID-19) Dashboard With Vaccination Data

For peer review only - http://bmjopen.bmj.com/site/about/guidelines.xhtml

2022 [Available from: https://covid19.who.int/region/afro/country/ug.

16. Africa CDC. COVID-19 vaccine perceptions: a 15-country study. : Africa CDC, 2021.

17. Schmid P, Rauber D, Betsch C, et al. Barriers of influenza vaccination intention and behavior–a systematic review of influenza vaccine hesitancy, 2005–2016. *PloS one* 2017;12(1):e0170550.

18. Betsch C, Böhm R, Korn L. Inviting free-riders or appealing to prosocial behavior? game-theoretical reflections on communicating herd immunity in vaccine advocacy. *Health Psychology* 2013;32(9):978.

19. World Health Organization. Behavioural considerations for acceptance and uptake of COVID-19 vaccines: WHO technical advisory group on behavioural insights and sciences for health, meeting report, 15 October 2020. 2020

20. MacDonald NE. Vaccine hesitancy: Definition, scope and determinants. *Vaccine* 2015;33(34):4161-64.

21. Sadique MZ, Devlin N, Edmunds WJ, et al. The effect of perceived risks on the demand for vaccination: results from a discrete choice experiment. *PloS one* 2013;8(2):e54149.

22. Dubé E, Laberge C, Guay M, et al. Vaccine hesitancy: an overview. *Human vaccines & immunotherapeutics* 2013;9(8):1763-73.

23. Betsch C, Böhm R, Chapman GB. Using behavioral insights to increase vaccination policy effectiveness. *Policy Insights from the Behavioral and Brain Sciences* 2015;2(1):61-73.

24. Echoru I, Ajambo PD, Keirania E, et al. Sociodemographic factors associated with acceptance of COVID-19 vaccine and clinical trials in Uganda: a cross-sectional study in western Uganda. *BMC Public Health* 2021;21(1):1106. doi: 10.1186/s12889-021-11197-7

25. McAbee L, Tapera O, Kanyangarara M. Factors associated with COVID-19 vaccine intentions in Eastern Zimbabwe: A cross-sectional study. *Vaccines* 2021;9(10):1109.

26. Moola S, Gudi N, Nambiar D, et al. A rapid review of evidence on the determinants of and strategies for COVID-19 vaccine acceptance in low-and middle-income countries. *Journal of global health* 2021;11

27. Adebisi YA, Alaran AJ, Bolarinwa OA, et al. When it is available, will we take it? Social media users' perception of hypothetical COVID-19 vaccine in Nigeria. *Pan Afr Med J* 2021;38

For peer review only - http://bmjopen.bmj.com/site/about/guidelines.xhtml

28. Abedin M, Islam MA, Rahman FN, et al. Willingness to vaccinate against COVID-19 among Bangladeshi adults: Understanding the strategies to optimize vaccination coverage. *PLoS One* 2021;16(4):e0250495.

29. Otiti-Sengeri J, Andrew OB, Lusobya RC, et al. High COVID-19 Vaccine Acceptance among Eye Healthcare Workers in Uganda. *Vaccines* 2022;10(4):609.

30. Muhamadi L, Edith N, James W, et al. Lack of Trust, Insufficient knowledge and Risk denial; an in-depth Understanding of Health worker Barriers to uptake of the Covid-19 vaccine at Iganga Hospital Eastern Uganda, and Mengo Hospital Kampala Uganda. *medRxiv* 2021

31. Paterson P, Meurice F, Stanberry LR, et al. Vaccine hesitancy and healthcare providers. *Vaccine* 2016;34(52):6700-06.

32. Uganda Bureau of Statistics (UBOS). Uganda National Household Survey 2019/2020. Kampala, Uganda: UBOS, 2021.

For peer review only - http://bmjopen.bmj.com/site/about/guidelines.xhtml

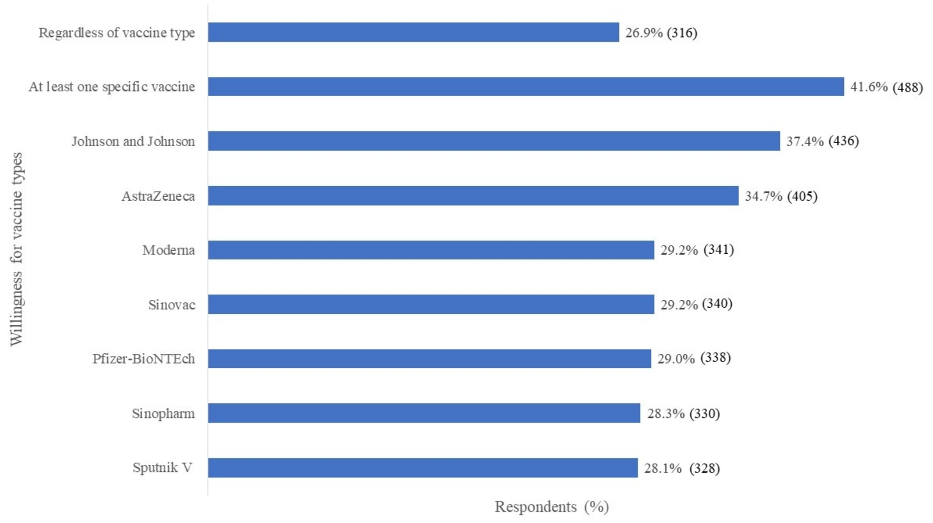

Willingness for COVID-19 vaccination for different vaccine types

338x190mm (96 x 96 DPI)

Uptake of COVID-19 vaccines and associated factors among adults in Uganda: a cross-sectional survey

Question	Answer	
MAKERERE SCHOOL OF PUBLIC HEALTH AND MIT GOV/LAB MOBILE SURVEY QUESTIONNAIRE		
Phone number used for interview		
Did the call go through?	1	Yes
	0	No
	-97	DO NOT READ: Refused
Was this the respondent previously interviewed?	1	Yes
	0	No
	-97	DO NOT READ: Refused
[Insert Consent Text]		
Do you voluntarily agree to participate in this survey?	1	Yes
	0	No
	-97	DO NOT READ: Refused
Explain:		
A7. What is your name?		
A8. What is your age?		
A9. What region do you live in?	1	North
	2	East
	3	Central
	4	West
	-97	DO NOT READ: Refused
A10. What district do you live in?	[Insert filtered list of districts]	
A11. Do you live in an urban, rural, or semi-urban area?	1	Urban
	2	Rural
	3	Semi-urban
	-99	DO NOT READ: Don't know
	-97	DO NOT READ: Refused
A12. Gender of respondent	1	Female
	0	Male
Next, I am going to ask some questions on your awareness and intention to take the COVID-19 vaccine.		
E1. As an adult, have you received a vaccine other than the COVID-19 vaccines? (Hepatitis B, Yellow fever, tetanus)	1	Yes
	0	No
	-97	DO NOT READ: Refused
E2. Have you ever been asked to get a vaccine and declined?	1	Yes
	0	No
	-97	DO NOT READ: Refused

Question	Answer	
E3. What COVID-19 vaccines have you heard of?<br>*Do not read options aloud. Select all that apply.*	1	AstraZeneca
	2	Pfizer-BioNTEch
	3	Moderna
	4	Johnson and Johnson
	5	Sinopharm
	6	Sinovac
	7	Sputnik V
	-96	Other (Specify)
	8	Has heard of COVID-19 vaccine but does not know any names
	0	None, has not heard of any COVID-19 vaccine
	-99	DO NOT READ: Don't Know
	-97	DO NOT READ: Refused
Other, specify:		
E4. Have you received the COVID-19 vaccine?<br>*Read all options aloud*	1	Yes, full dose
	2	Yes, incomplete dose
	0	No
	-97	DO NOT READ: Refused
E5. Which vaccine did you receive?<br>*Do not read options aloud. Select all that apply.*	1	AstraZeneca
	2	Pfizer-BioNTEch
	3	Moderna
	4	Johnson and Johnson
	5	Sinopharm
	6	Sinovac
	7	Sputnik V
	-96	Other (Specify)
	8	Has heard of COVID-19 vaccine but does not know any names
	0	None, has not heard of any COVID-19 vaccine
	-99	DO NOT READ: Don't Know
	-97	DO NOT READ: Refused
Other, specify:		

Question	Answer	
E6. Why did you receive the COVID-19 vaccine?<br>*Do not read options aloud. Select all that apply.*	1	To protect self / others from COVID-19
	2	High perceived risk of getting COVID-19
	3	Travel purposes
	4	Recommendation from health workers
	5	Prioritized due to occupation
	6	Prioritized due to health
	-96	Other (Specify)
	-99	DO NOT READ: Don't Know
	-97	DO NOT READ: Refused
Other, specify:		
E7. Did you experience any side effects within 7 days following the first dose?	1	Yes
	0	No
	-97	DO NOT READ: Refused
E8. Which side effects did you experience following the first dose?<br>*Do not read options aloud. Select all that apply.*	1	Fever
	2	Fatigue
	3	Headache
	4	Muscle soreness/pain (myalgia)
	5	Allergic reaction
	6	Injection site reaction
	-96	Other (Specify)
	-97	DO NOT READ: Refused
Other, specify:		
E9. Did you experience any side effects within 7 days following the second dose?	1	Yes
	0	No
	-97	DO NOT READ: Refused
E10. Which side effects did you experience following the second dose?<br>*Do not read options aloud. Select all that apply.*	1	Fever
	2	Fatigue
	3	Headache
	4	Muscle soreness/pain (myalgia)
	5	Allergic reaction
	6	Injection site reaction
	-96	Other (Specify)
	-97	DO NOT READ: Refused
Other, specify:		

Question	Answer		
E11. Why haven't you ever received the COVID-19 vaccine?<br>*Do not read options aloud. Select all that apply.*		1	Safety concerns / fear of adverse events
		2	Doubt vaccine effectiveness
		3	Do not fear COVID-19 / trust immunity
		4	Don't know where to access vaccine from
		5	Religious beliefs
		6	COVID-19 is a hoax / politics
		7	Not among eligible group
		8	Not having time
		9	Transport costs
		10	Cost of vaccine
		11	Vaccines are unavailable
		-96	Other (Specify)
		-99	DO NOT READ: Don't know
		-97	DO NOT READ: Refused
Other, specify:			
E12. Do you intend to receive the COVID-19 vaccine if it is available?		1	Yes
		0	No
		-97	DO NOT READ: Refused
E13. Why do you intend to receive the COVID-19 vaccine?<br>*Do not read options aloud. Select all that apply.*		1	To protect self / others from COVID-19
		2	High perceived risk of getting COVID-19
		3	Travel purposes
		4	Recommendation from health workers
		5	Prioritized due to occupation
		6	Prioritized due to health
		-96	Other (Specify)
		-99	DO NOT READ: Don't Know
		-97	DO NOT READ: Refused
Other, specify:			
E14. Why don’t you intend to receive the COVID-19 vaccine?<br>*Do not read options aloud. Select all that apply.*		1	Safety concerns / fear of adverse events
		2	Doubt vaccine effectiveness

Question	Answer	
	3	Do not fear COVID-19 / trust immunity
	4	Don't know where to access vaccine from
	5	Religious beliefs
	6	COVID-19 is a hoax / politics
	7	Not among eligible group
	8	Not having time
	9	Transport costs
	10	Cost of vaccine
	11	Vaccines are unavailable
	-96	Other (Specify)
	-99	DO NOT READ: Don't know
	-97	DO NOT READ: Refused
Other, specify:		
E15. Has anyone in your household received their COVID-19 vaccine?	1	Yes, specify
	0	No
	-99	DO NOT READ: Don't Know
	-97	DO NOT READ: Refused
E16. In your household, who has received their COVID-19 vaccine? *Do not read options aloud. Select all that apply.*	1	Parents
	2	Children
	3	Grandparents
	4	Grandchildren
	5	Siblings
	6	Cousins
	7	Aunts and Uncles
	8	Spouse
	-96	Other (Specify)
	-97	DO NOT READ: Refused
Other, specify:		
E17. Do you know anyone personally in your community who has received their COVID-19 vaccine?	1	Yes, specify
	0	No
	-99	DO NOT READ: Don't Know
	-97	DO NOT READ: Refused

Question		Answer
E18. Among those you know personally in your community, who has received their COVID-19 vaccine?<br>*Do not read options aloud. Select all that apply.*	1	Traditional leaders
	2	Religious leaders
	3	Colleagues
	4	Personal friends
	5	Neighbors
	-96	Other (Specify)
	-97	DO NOT READ: Refused
Other, specify:		
Next, I am going to ask some questions about COVIDEX.		
E19. Have you ever heard of COVIDEX before?	1	Yes
	0	No
	-97	DO NOT READ: Refused
E20. Has anyone in your household ever bought or used COVIDEX?	1	Yes
	0	No
	-99	DO NOT READ: Don't Know
	-97	DO NOT READ: Refused
E21. Personally, how much trust do you have in COVIDEX?<br>*Read all options aloud*	1	Trust it a great deal
	2	Tend to trust it
	3	Tend to distrust it
	4	Distrust it greatly
	-99	DO NOT READ: Not sure or don't know
	-97	DO NOT READ: Refused
Next, I am going to ask some more questions on COVID-19 vaccines.		
(Randomize order of F2)		
F2. If you were offered the COVID-19 **AstraZeneca** vaccine right now free of cost, would you take the vaccine?<br>*Read all options aloud*	1	Yes
	0	No
	-95	Have not heard of
	-97	DO NOT READ: Refused
F2. If you were offered the COVID-19 **Pfizer-BioNTEch** vaccine right now free of cost, would you take the vaccine?<br>*Read all options aloud*	1	Yes
	0	No
	-95	Have not heard of
	-97	DO NOT READ: Refused
F2. If you were offered the COVID-19 **Moderna** vaccine right now free of cost, would you take the vaccine?<br>*Read all options aloud*	1	Yes
	0	No
	-95	Have not heard of
	-97	DO NOT READ: Refused
F2. If you were offered the COVID-19 **Johnson and Johnson** vaccine right now free of cost, would you take the vaccine?<br>*Read all options aloud*	1	Yes
	0	No
	-95	Have not heard of
	-97	DO NOT READ: Refused

Question	Answer	
F2. If you were offered the COVID-19 **Sinopharm** vaccine right now free of cost, would you take the vaccine?<br>*Read all options aloud*	1	Yes
	0	No
	-95	Have not heard of
	-97	DO NOT READ: Refused
F2. If you were offered the COVID-19 **Sinovac** vaccine right now free of cost, would you take the vaccine?<br>*Read all options aloud*	1	Yes
	0	No
	-95	Have not heard of
	-97	DO NOT READ: Refused
F2. If you were offered the COVID-19 **Sputnik V** vaccine right now free of cost, would you take the vaccine?<br>*Read all options aloud*	1	Yes
	0	No
	-95	Have not heard of
	-97	DO NOT READ: Refused
F3. Why would you choose these vaccines?	1	More effective
	2	Less adverse events
	3	Used in Western countries
	4	Trust in source
	-96	Other (Specify)
	-99	DO NOT READ: Don't Know
	-97	DO NOT READ: Refused
Other, specify		
F4. If you needed information on vaccines, who in your community would you talk to for advice?		
F5. What are your sources of information on COVID-19?<br>*Do not read options aloud. Select all that apply.*	1	Family members
	2	Friends/peers
	3	Health worker
	4	Phone (messages and calls)
	5	Radio
	6	Television
	7	Church/Mosque
	8	Community member/village health team member
	9	Local leader
	10	Social media (Facebook, WhatsApp, Twitter)
	11	Internet
	-96	Other (Specify)
	-99	DO NOT READ: Don't Know
	-97	DO NOT READ: Refused

Question	Answer	
Other, specify		
F6. Which three sources of information do you trust the most?<br>*Do not read options aloud. Select all that apply.*	1	Family members
	2	Friends/peers
	3	Health worker
	4	Phone (messages and calls)
	5	Radio
	6	Television
	7	Church/Mosque
	8	Community member/village health team member
	9	Local leader
	10	Social media (Facebook, WhatsApp, Twitter)
	11	Internet
	-96	Other (Specify)
	-99	DO NOT READ: Don't Know
	-97	DO NOT READ: Refused
Other, specify		
F7. Have you ever been tested at a health facility or laboratory and found to have COVID-19?	1	Yes
	0	No
	-97	DO NOT READ: Refused
F8. Was this COVID infection before or after you received the full dose of the vaccine?	1	Before
	2	After
	3	Both
	-97	DO NOT READ: Refused
B1. What is your highest level of education?	1	No school
	2	Some primary
	3	Complete primary
	4	Secondary - ordinary
	5	Secondary - advanced
	6	Tertiary
	-96	Other (Specify)
	-97	DO NOT READ: Refused
Other, specify		
B2. What is your religion?	1	Catholic
	2	Anglican
	3	Born Again/Pentecostal
	4	Muslim
	-96	Other (Specify)
	-97	DO NOT READ: Refused

Question	Answer	
Other, specify		
B3. On average, how much money do you earn per month?<br>*In Ugandan Shillings*	1	under 50,000
	2	50,001 - 100,000
	3	100,001 - 200,000
	4	200,001 - 500,000
	5	500-001 - 1,000,000
	6	1,000,001 and above
	-97	DO NOT READ: Refused
B4. Does your household have **a television**?	1	Yes
	0	No
	-99	DO NOT READ: Don't Know
	-97	DO NOT READ: Refused
B4. Does your household have **electricity**?	1	Yes
	0	No
	-99	DO NOT READ: Don't Know
	-97	DO NOT READ: Refused
B4. Does your household have **a computer**?	1	Yes
	0	No
	-99	DO NOT READ: Don't Know
	-97	DO NOT READ: Refused
B4. Does your household have a **sofa set**?	1	Yes
	0	No
	-99	DO NOT READ: Don't Know
	-97	DO NOT READ: Refused
B4. Does your household have a **refrigerator**?	1	Yes
	0	No
	-99	DO NOT READ: Don't Know
	-97	DO NOT READ: Refused
B5. Does anyone in your household own a **cassette/CD/DVD player**?	1	Yes
	0	No
	-99	DO NOT READ: Don't Know
	-97	DO NOT READ: Refused

B6A. What is your current occupation?<br>*Read all options aloud*	1	Unemployed/retiree/housewife
	2	Employed
	3	Self-employed
	4	Casual laborer
	5	Farmer
	-96	Other (Specify)
	-97	DO NOT READ: Refused
Other, specify:		
B7A. How many people stay in your home, currently?		

For peer review only

STROBE Statement—Checklist of items that should be included in reports of ***cross-sectional studies***

	Item No	Recommendation	Page No
**Title and abstract**	1	(*a*) Indicate the study's design with a commonly used term in the title or the abstract	1
		(*b*) Provide in the abstract an informative and balanced summary of what was done and what was found	1
**Introduction**			
Background/rationale	2	Explain the scientific background and rationale for the investigation being reported	4,5
Objectives	3	State specific objectives, including any prespecified hypotheses	5
**Methods**			
Study design	4	Present key elements of study design early in the paper	5
Setting	5	Describe the setting, locations, and relevant dates, including periods of recruitment, exposure, follow-up, and data collection	5
Participants	6	(*a*) Give the eligibility criteria, and the sources and methods of selection of participants	5
Variables	7	Clearly define all outcomes, exposures, predictors, potential confounders, and effect modifiers. Give diagnostic criteria, if applicable	6
Data sources/ measurement	8*	For each variable of interest, give sources of data and details of methods of assessment (measurement). Describe comparability of assessment methods if there is more than one group	*6*
Bias	9	Describe any efforts to address potential sources of bias	6,20
Study size	10	Explain how the study size was arrived at	6
Quantitative variables	11	Explain how quantitative variables were handled in the analyses. If applicable, describe which groupings were chosen and why	7,8
Statistical methods	12	(*a*) Describe all statistical methods, including those used to control for confounding	7,8
		(*b*) Describe any methods used to examine subgroups and interactions	7,8
		(*c*) Explain how missing data were addressed	7,8
		(*d*) If applicable, describe analytical methods taking account of sampling strategy	Not applicable
		(*e*) Describe any sensitivity analyses	Not applicable
**Results**			
Participants	13*	(a) Report numbers of individuals at each stage of study—eg numbers potentially eligible, examined for eligibility, confirmed eligible, included in the study, completing follow-up, and analysed	9
		(b) Give reasons for non-participation at each stage	9
		(c) Consider use of a flow diagram	Not necessary
Descriptive data	14*	(a) Give characteristics of study participants (eg demographic, clinical, social) and information on exposures and potential confounders	9

For peer review only - http://bmjopen.bmj.com/site/about/guidelines.xhtml

		(b) Indicate number of participants with missing data for each variable of interest	9-16
Outcome data	15*	Report numbers of outcome events or summary measures	9-16
Main results	16	(*a*) Give unadjusted estimates and, if applicable, confounder-adjusted estimates and their precision (eg, 95% confidence interval). Make clear which confounders were adjusted for and why they were included	9-16
		(*b*) Report category boundaries when continuous variables were categorized	9-16
		(*c*) If relevant, consider translating estimates of relative risk into absolute risk for a meaningful time period	Not relevant
Other analyses	17	Report other analyses done—eg analyses of subgroups and interactions, and sensitivity analyses	None
**Discussion**			
Key results	18	Summarise key results with reference to study objectives	17
Limitations	19	Discuss limitations of the study, taking into account sources of potential bias or imprecision. Discuss both direction and magnitude of any potential bias	20,21
Interpretation	20	Give a cautious overall interpretation of results considering objectives, limitations, multiplicity of analyses, results from similar studies, and other relevant evidence	17-21
Generalisability	21	Discuss the generalisability (external validity) of the study results	20,21
**Other information**			
Funding	22	Give the source of funding and the role of the funders for the present study and, if applicable, for the original study on which the present article is based	22

*Give information separately for exposed and unexposed groups.

Note: An Explanation and Elaboration article discusses each checklist item and gives methodological background and published examples of transparent reporting. The STROBE checklist is best used in conjunction with this article (freely available on the Web sites of PLoS Medicine at http://www.plosmedicine.org/, Annals of Internal Medicine at http://www.annals.org/, and Epidemiology at http://www.epidem.com/). Information on the STROBE Initiative is available at www.strobe-statement.org.

For peer review only
